# Titanium Nitride Coatings on CoCrMo and Ti6Al4V Alloys: Effects on Wear and Ion Release

**DOI:** 10.3390/lubricants12030096

**Published:** 2024-03-15

**Authors:** Mohammed AbuAlia, Spencer Fullam, Filippo Cinotti, Noora Manninen, Markus A. Wimmer

**Affiliations:** 1 Department of Orthopedics, Rush University Medical Center, Chicago, IL 60612, USA; 2 Oerlikon Surface Solutions AG, 9496 Balzers, Liechtenstein

**Keywords:** orthopedic implants, titanium nitride coating, wear resistance, ion release, CoCrMo alloy, Ti6Al4V alloy, UHMWPE, advanced coating processes

## Abstract

While titanium nitride (TiN) coatings are well known for their biocompatibility and excellent mechanical properties, their wear particle and debris release in orthopedic implants remains a matter of active investigation. This study addresses the efficacy of TiN coatings on CoCrMo and Ti6Al4V alloys to enhance wear resistance and reduce ion release from prosthetic implants. Three different coating variants were utilized: one variant deposited using arc evaporation (Arc) followed by post-treatment, and two variants deposited using high-power impulse magnetron sputtering (HiPIMS) with or without post-treatment. The coatings’ performance was assessed through standard wear testing against ultra-high-molecular-weight polyethylene (UHMWPE) in bovine serum lubricant, and in the presence of abrasive PMMA bone cement particles in the lubricant. The results indicated that Arc and HiPIMS with post-treatment significantly reduced wear and eliminated detectable metal ion release, suggesting that these coatings could extend implant longevity and minimize adverse biological responses. Further long-term simulator and in vivo studies are recommended to validate these promising findings.

## 1. Introduction

The advent of total joint replacements (TJRs) has significantly improved outcomes for patients with debilitating joint diseases, yet their long-term success is challenged by the occurrence of wear particles and debris [1]. These by-products are significant contributors to aseptic loosening and wear or osteolysis, which are among the leading causes of implant failure and subsequent revision surgeries. The *AJRR 2023 Annual Report* [2] provides insight into the distribution of diagnoses associated with all hip and knee revisions from 2012 to 2022. Aseptic loosening accounts for 19.71% of hip revisions, and wear or osteolysis accounts for 6.94%, highlighting the notable impact of these factors on hip implant longevity. Similarly, wear or osteolysis is also a concern in knee revisions, representing 3.9% of the cases, emphasizing the importance of understanding and mitigating these conditions to improve implant survival rates. Research indicates that wear particles in the periprosthetic microenvironment trigger a chronic inflammatory response, which can culminate in aseptic loosening. The innate immune response activated by these particles involves key immune cells, which produce inflammatory cytokines and chemokines, exacerbating the condition. This underscores the need for continued efforts to minimize wear particle production to prolong the lifespan of implants and reduce the risk of aseptic loosening [3].

Ultra-high-molecular-weight polyethylene (UHMWPE) wear particles in particular initiate osteolytic processes [4,5]. The presence of PMMA bone cement particles exacerbates the wear rates, further highlighting the seriousness of “the wear problem” in orthopedic implants [6]. The systemic migration of metal ions from the debris poses additional risks, including accumulation in vital organs such as the liver, kidneys, heart, and brain [7,8].

In response to these challenges, the surface modification of implant materials has been recognized as a critical factor in influencing wear production. Advances in coating technologies have aimed to enhance the surface properties of implants, preserving the mechanical integrity of the substrate material [9]. Titanium nitride (TiN) has emerged as a promising candidate for coating materials due to its low friction coefficient, high hardness and wear resistance, excellent biocompatibility, and chemical stability, making it suitable for orthopedic applications [10–12]. The comparative fretting corrosion performance of Ti6Al4V when paired with CoCrMo, as studied by Swaminathan and Gilbert, reinforces TiN’s potential advantages, demonstrating that such coatings may help mitigate the complex electrochemical and mechanical interactions at the material interfaces, which are crucial for implant longevity [13].

Despite the known benefits of titanium nitride (TiN) coatings, their application in total knee and hip implants has been cautious, largely due to potential issues like coating delamination and the generation of ceramic wear particles. Van Hove et al.’s [14] systematic review in 2015 pinpointed to complications including delamination, escalated UHMWPE wear, as well as cohesive failure, highlighting the dependency of these outcomes on coating process specifics and substrate selection. Building on these concerns, a 2022 review and meta-analysis corroborated that while TiN coatings may match uncoated implants in mid-term performance, their long-term reliability critically hinges on the durability of the coating–substrate bond and resilience to the demanding joint environment, emphasizing the vital role of advanced deposition methods and post-coating treatments in boosting wear resistance and curbing the release of particulates [15].

In response to recent findings, our study has incorporated advanced physical vapor deposition (PVD) techniques, specifically arc evaporation (Arc) and high-power impulse magnetron sputtering (HiPIMS), to enhance the quality of coatings in orthopedic implants. Arc technology, known for producing robust and adherent coatings, often necessitates post-treatment due to microdroplet-induced roughness, a challenge acknowledged in prior studies [16,17]. This post-treatment is crucial to rectify the significant defects and high defect density observed in TiN coatings applied via Arc technology, especially as seen in the retrieval analysis of coated prosthetic femoral heads [16].

Conversely, HiPIMS is renowned for its ability to produce inherently smoother coatings with superior density and corrosion resistance, which are key traits for the longevity of orthopedic implants [18]. The advancements in HiPIMS technology, especially in the deposition of CrN/NbN and Cr2AlC MAX phase coatings, indicate its capacity to meet and exceed load requirements while reducing metal ion release [19,20]. Additionally, the exploration of HiPIMS W-C and W-C:H coatings in tribological applications suggests the potential for achieving superlubricity under certain conditions, a testament to its suitability for orthopedic applications [21,22]. Despite its advantages, our study indicates that post-polishing for HiPIMS coatings might still be necessary, but this technology opens avenues for further investigation into long-term performance benefits over Arc methods.

The evolution of implant material selection underpins the rationale for these advanced coating techniques. Initially, titanium was favored for its biocompatibility, but its susceptibility to wear under abrasive conditions led to a shift towards cobalt chromium alloys for articulating metals [22,23]. However, not all patients can tolerate these alloys, and their use has been linked to issues like host bone resorption [24–26]. Coatings, particularly those developed through micro-arc oxidation, have shown promise in improving not only the osteogenic, antibacterial, and anti-inflammatory properties of metal implants [27–30], but also their wear properties [29,31,32], underscoring the significance of advanced coating technologies in addressing these challenges.

Our study aims to provide a general understanding of the efficacy of these advanced surface modification techniques in improving wear properties of TiN-coated titanium and cobalt-chromium surfaces against UHMWPE. It encompasses an evaluation of novel coating technologies, especially HiPIMS, against established Arc-PVD methods in a bio-tribological setting. The effects of their capacity to withstand abrasive forces from elements like PMMA bone cement particles is also investigated. Our rigorous testing procedure, governed by the stringent conditions of multidirectional pin-on-disk wear trials, offers crucial insights for selecting coating materials and methods. This methodology bridges the gap between lab-scale testing and expected in vivo performance, considering that coatings, though resilient to delamination in scratch tests, may succumb to the harsh environment within the body [33,34].

## 2. Materials and Methods

### 2.1. Testing Materials and Substrates

**Figure 1.**
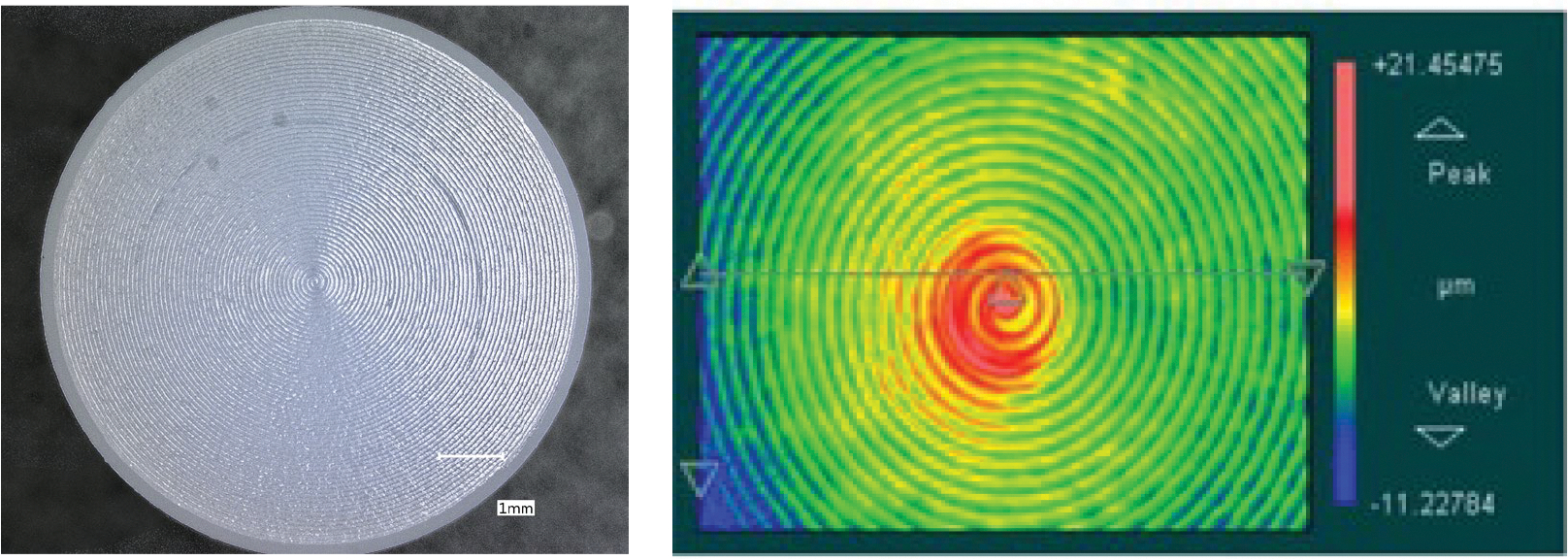
Visualization of an untested UHMWPE pin surface. The left image, obtained through light microscopy at a scale bar of 1 mm, showcases the original machining marks. The right image displays a surface map generated using white light interferometry, capturing the topographical contours with peak-to-valley measurements.

**Figure 2.**
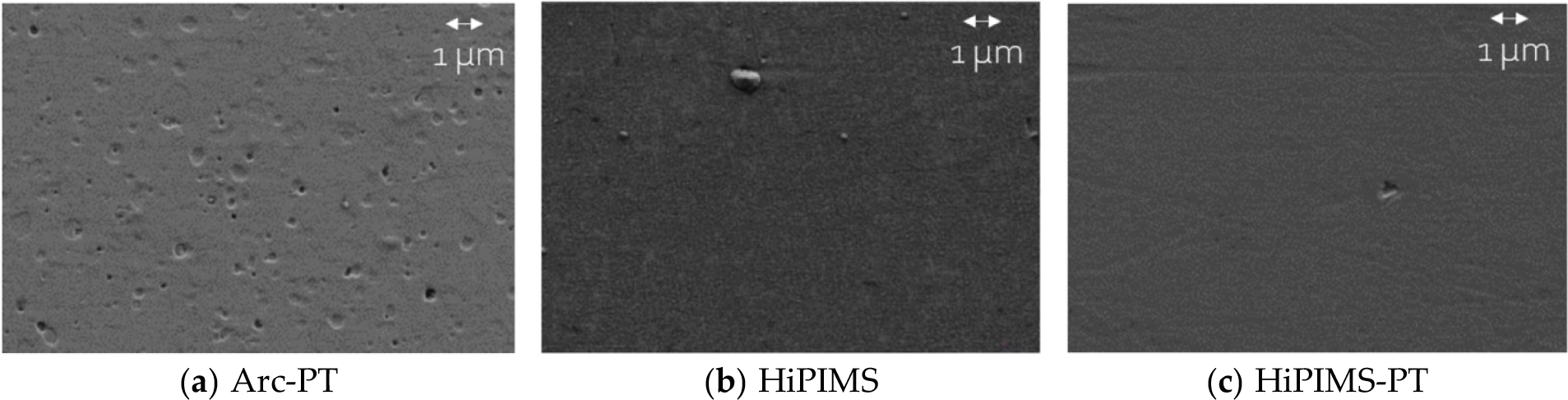
Surface morphology of TiN coatings (for further explanations, see Section 2.2). (**a**) illustrates the arc evaporation (Arc-PT) coating after post-treatment, where the initial microdroplets have been transformed into depressions, which are characteristic consequences of the post-treatment process. (**b**) depicts the HiPIMS coated surface in its as-deposited condition, with only a few small, raised droplets visible. (**c**) shows the HiPIMS-PT surface, which has undergone post-treatment. Note the presence of a single small depression. Scale bar 1 micrometer.

Discs made of CoCrMo (delivered according to ASTM F1537 [35] in warm-worked condition) and Ti6Al4V alloy (additively manufactured from powder fulfilling ASTM F1108 [36] and F2924 [37], i.e., stress relieved and HIPed), each measuring 12.62 mm in height and 43.50 mm in diameter, were obtained with a mirror-like surface finish from local material suppliers (Fein und Präzisionmechanik AG, Switzerland, resp. Oerlikon AM GmbH, Germany) for TiN coating applications by Oerlikon (Balzers, Liechtenstein). Additionally, 61 cylindrical UHMWPE pins (GUR 1050), treated with 30 ± 5 kGy radiation to sterilize and to induce conventional crosslinking, were purchased from Orthoplastics (Lancashire, UK), with dimensions of 19.89 mm in height and 10.50 mm in diameter. The surface topography of an untested UHMWPE pin is depicted in Figure 1. Figure 2 showcases the surface topography of titanium nitride (TiN) coatings applied on alloy discs, highlighting the effects of different deposition methods and post-treatment on the surface morphology. The average surface roughness (R_a_) values for both coated and uncoated discs, as received, are detailed in Table 1.

### 2.2. Coating Deposition and Characterization

Single-layer titanium nitride (TiN) coatings, i.e., without the use of any interlayer between the substrate and the TiN coating, were applied to CoCrMo and Ti6Al4V alloy discs using two different coating technologies, namely, arc evaporation (Arc) and high-power impulse magnetron sputtering (HiPIMS). These methods, which are proprietary variants of the physical vapor deposition (PVD) process, were originally developed to improve wear resistance and biocompatibility critical to orthopedic implants [15].

Arc evaporation is a high ionization technique that utilizes a low-voltage, high-current arc to melt and vaporize the target material, which then condenses on the substrate, forming a dense and hard coating with strong adhesion properties. A potential downside is the production of microdroplets, which can roughen the coating’s surface [19–21] and must be removed during the post-treatment of the surface. In this study, the TiN arc coating was deposited through arc evaporation using high purity Ti targets evaporated in an N_2_ atmosphere. The working pressure in the deposition chamber was 3.5 Pa, with a target current density maintained at 0.8 A/cm^2^. The coating was deposited at 200 °C, with a bias voltage of −100 V applied to the substrate holder. Prior to coating deposition, an in situ plasma etching in an Ar + H_2_ atmosphere was performed to ensure good coating adhesion.

HiPIMS, on the other hand, is a sputtering-based ionized physical vapor deposition technique which strongly reduces the generation of microdroplets. The main difference in relation to conventional magnetron sputtering processes is the mode of operation: in HiPIMS, the power is applied to the target in unipolar pulses at a low duty factor (<10%) and low frequency (<10 kHz), leading to peak target power densities in the order of several kilowatts per square centimeter. The high power of the process allows the production of coatings with high density and hardness, similar to arc coatings but with a lower density of droplets. The TiN HiPIMS coating was deposited using high purity Ti targets in a N_2_ + Ar atmosphere. The working pressure in the deposition chamber was maintained at 0.45 Pa, with a target power density of 55 W/cm^2^. The coatings were deposited at 400 °C, with a bias voltage of −50 V. Similar to the arc process, an in situ plasma etching in an Ar + H_2_ atmosphere was performed prior to coating deposition to ensure adhesion.

In this study, both coating variants were submitted to a post-treatment process which consists of a microblasting process where the coated parts are submitted to an abrasive jet of microparticles which allow the removal of droplets from the coated parts. In order to remove any possible remaining particles resulting from the microblasting process of the coated samples, a solvent-based cleaning process was used. The HiPIMS coating variant was tested in the as-deposited condition (i.e., without post-treatment), and after post-treatment.

### 2.3. Thickness Evaluation and Coating Hardness

Ball crater tests, following ISO 26423:2009 [38], were used to measure the film thickness. The coatings exhibited the following thicknesses: Arc PT (post-treatment) at 4 μm, HiPIMS at 5 μm, and HiPIMS with post-treatment (HiPMS-PT) at 5 μm.

The hardness of the coatings was evaluated using a Fischerscope system, in compliance with ISO 14577 [39]. The system, equipped with a Berkovich indenter and a maximum load of 10 mN, resulted in an indentation depth of approximately 10% of the film thickness. The hardness measurements were 30.3 ± 1.3 GPa for the Arc PT, 33.5 ± 1.8 GPa for HiPIMS, and 32 ± 0.8 GPa for HiPMS-PT (Table 2).

### 2.4. Wear Testing

#### 2.4.1. Pin-on-Disc Standard Wear Testing Using Bovine Serum

The wear testing was carried out using a six-station pin-on-disc testing apparatus (OrthoPod AMTI^®^, Boston, MA, USA), specifically designed to mimic the tribological environment of joint replacements. This system allows for the precise control of pin rotation, disc rotation, and loading force, with each station equipped to accommodate a cylindrical UHMWPE pin and a metal alloy disc, as described in detail previously [40]. In this study, a constant load of 200 N was used, translating to 2.8 MPa in contact pressure, typical for a knee or hip joint.

A total of three pairs of discs and pins were tested for each type of coated and uncoated substrate, running each pair for one million cycles (Mc). Prior to the commencement of each test round, the UHMWPE pins were pre-soaked in a bovine serum-based lubricant to ensure uniform fluid absorption. This step is critical as UHMWPE is known to absorb fluids, particularly under load, which can influence weight loss measurements. The pins underwent a vacuum desiccation process for 30 min and were then weighed with precision to establish a baseline mass. This process was repeated after soaking the pins in a 37 °C bath to ensure a consistent and stable weight, thereby allowing for an accurate assessment of wear by weight loss. This protocol ensures that any weight change observed post testing can be attributed to material wear and is not masked by fluid uptake.

**Figure 3.**
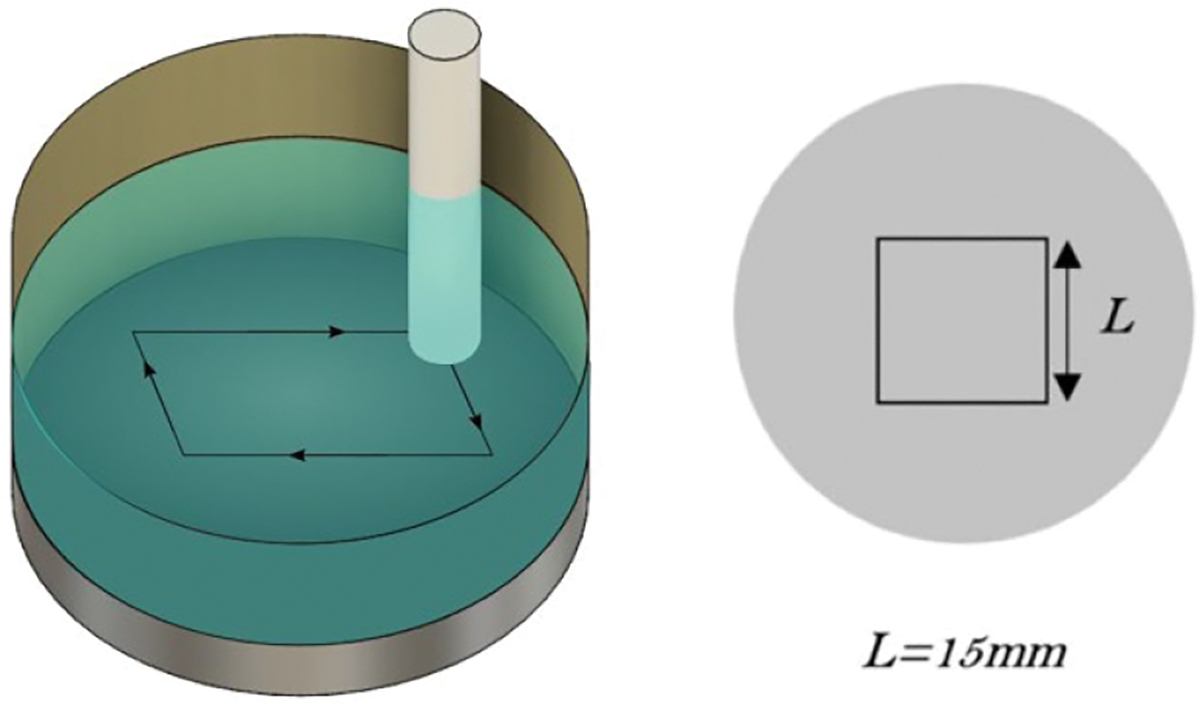
Three-dimensional isometric view of the wear test chamber along with a two-dimensional top view diagram detailing the motion pattern of the pin. On the left, the apparatus is shown with a cylindrical pin positioned above a disc submerged in a lubricant-filled container. The pin is aligned to follow a precise square trajectory on the disc’s surface, which is delineated by arrows indicating the direction of motion. The right side of the figure clarifies the motion pattern, with a square marked with the dimension ‘L’, indicating the length of each side of the square path, which is set to 15 mm.

During the test, the pins were subjected to a 15 mm × 15 mm square motion pattern on the discs to create wear tracks (Figure 3) at a rate of 1 Hz. This pattern is designed to generate crossing motion trajectories, which lead to higher wear rates and more accurately simulate the complex motions experienced by joint implants compared to unidirectional stress patterns [41].

The wear testing environment was carefully controlled to mimic bodily conditions. The articulating surfaces were submerged in a testing solution composed of bovine serum (NBCS Gibco^®^, Thermo Fisher Scientific, Waltham, MA, USA) with added deionized water and sodium chloride to reach a protein concentration of 30 g/L, adjusted to physiological salt levels. Using hydrochloric acid, the pH was adjusted to and maintained at 7.6 to replicate the slightly alkaline environment of the human body. A total of 27 mg/mL Tris buffer was used to prevent unwanted chemical reactions over time, alongside 0.2 mg/mL EDTA and antimicrobial agents (0.3% Sodium Azide and Penicillin Streptomycin) to avoid calcium precipitations and bacterial contamination. The temperature of the solution was kept constant at 37 °C, mirroring human body temperature.

Following the wear tests, cleaning procedures were stringently applied at regular intervals, at every 0.25 million cycles. Both metal and polymer components were ultrasonically cleaned using distilled water, Terg-A-Zyme^®^ (Alconox Inc., White Plains, NY, USA), and propanol, each for a duration of 10 min, and subsequently dried with a jet of N_2_ gas. Post cleaning, the pins were weighed to assess gravimetric wear. The weight loss per pin was calculated by averaging six separate measurements, in accordance with ASTM standards 2025 and F732, using a Labconco XPert^®^ Weigh Box (Kansas City, MS, USA). Any weight gain observed in soak control specimens, which were not subjected to wear but were immersed in the testing fluid, was subtracted from the total weight loss to correct for fluid absorption. This process allowed for the determination of an accurate linear wear rate, excluding the initial ‘run-in’ period where wear rates tend to be higher and more variable.

**Figure 4.**
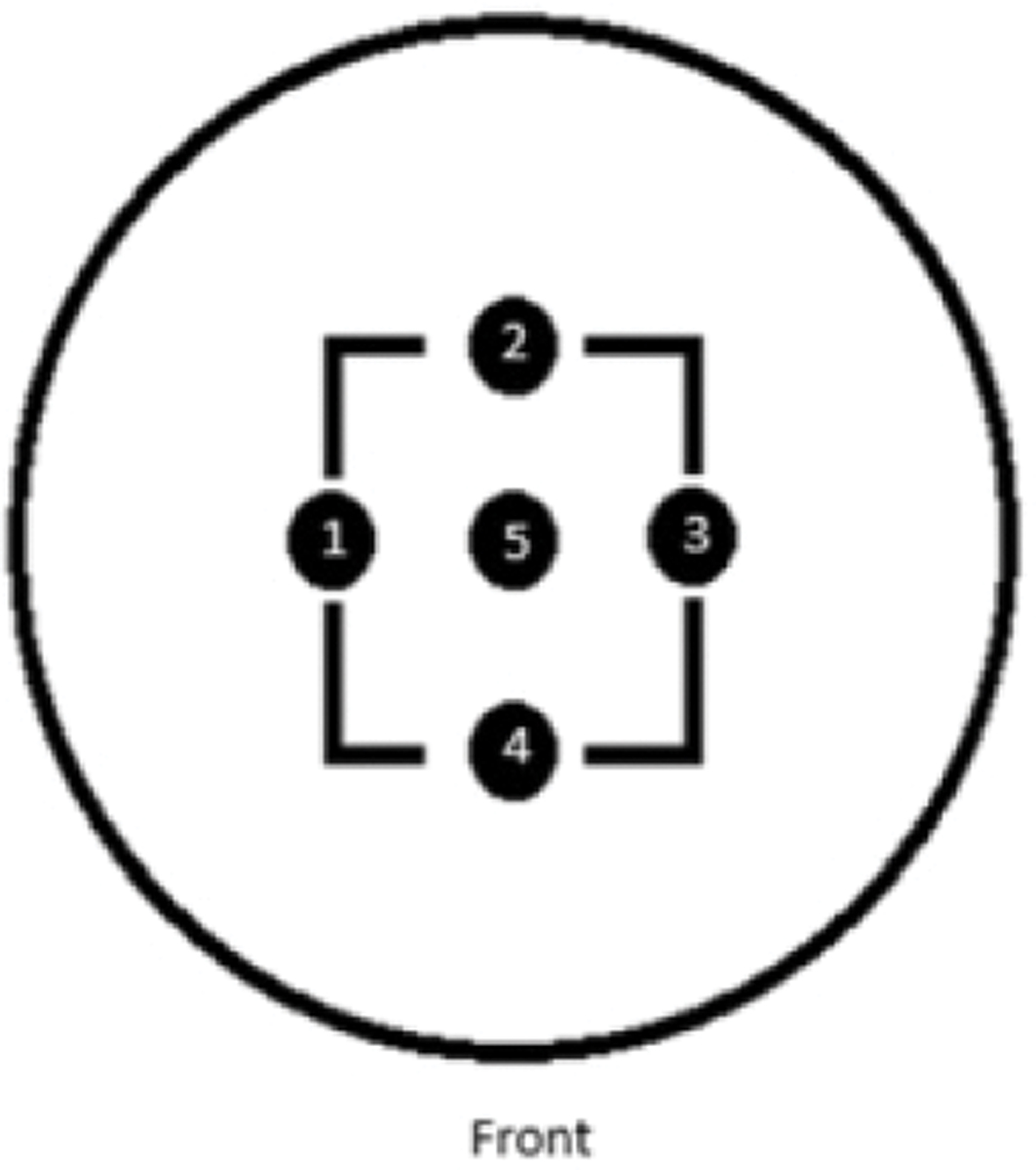
Schematic representation of the measurement mask utilized for assessing surface roughness on the wear tracks of the alloy discs. The diagram depicts a circular disc with a concentric square pattern marked by points 1 to 4, which designate the specific locations where roughness data were collected along the paths subjected to wear. Point 5, situated at the center of the square, indicates a region that was not exposed to wear, serving as a reference for the original surface condition. The term ‘Front’ at the bottom indicates the orientation of the disc relative to the observer during measurement.

The surface roughness of the orthopedic implant discs was characterized using a white light interferometer (Zygo^®^ Newview 6300, Middlefield, CT, USA). Measurements were conducted using a 20× magnification lens, and the MetroPro application was utilized for analysis. To ensure consistent and reproducible roughness measurements, a predefined mask was applied to the discs (Figure 4). This mask delineated four measurement points within the wear path and one on an unworn section in the center of the disc surface. Each point was carefully focused by adjusting the Zygo’s light intensity and orienting the interference fringes parallel to the bottom screen border with the device’s adjustment screws. To assess wear effects, ten measurements—five pre-test and five post test—were taken for each disc. This dual-phase measurement allowed for a comparative analysis of surface roughness alterations, providing a quantitative assessment of the changes in surface characteristics.

#### 2.4.2. Pin-on-Disc Wear Test Adding PMMA Bone Cement Particles

**Figure 5.**
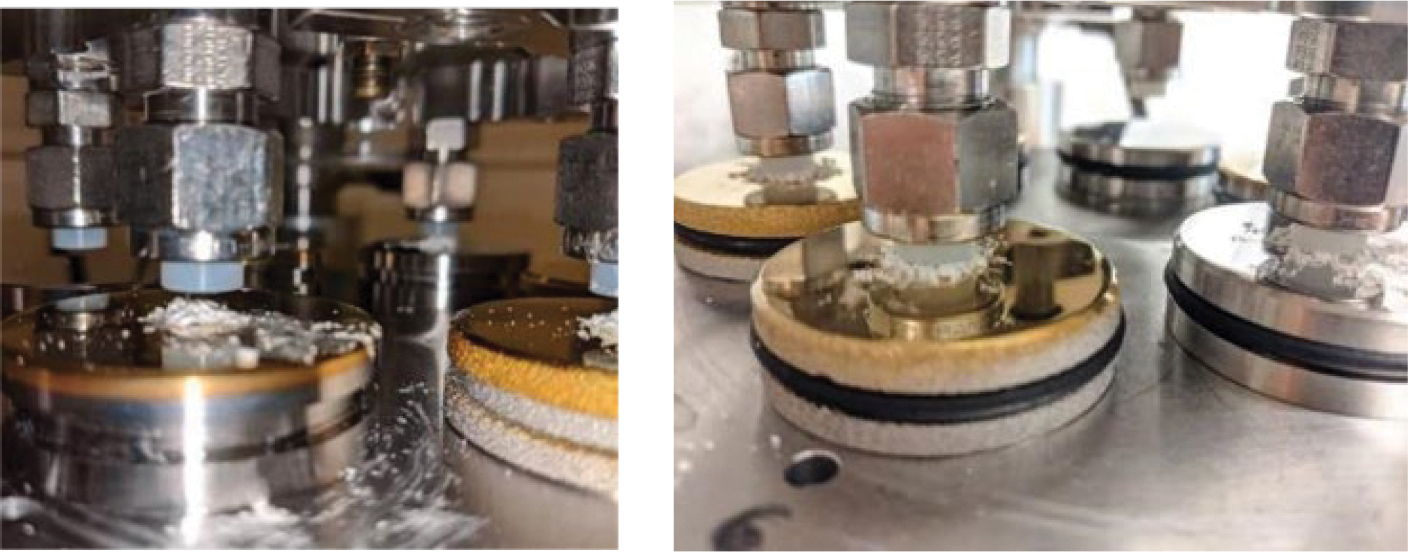
14 mg of PMMA particles were loaded below each UHMWPE pin. The pins were lowered and brought into contact with the PMMA particles. Running-in included an initial 1000-cycle dry run, followed by the repositioning of expelled particles and an additional 1000 cycles before the lubricant (described under Section 2.4.1) was introduced.

To examine the potentially protective behavior of TiN coatings in the presence of PMMA bone cement particles, the two most promising coatings on both metal substrates underwent continued testing against clinically relevant uncoated CoCrMo (Note: uncoated Ti6Al4V is not in clinical use). This secondary phase of testing introduced a challenge to the coatings with the inclusion of PMMA particles, prepared from polymerized Palacos bone cement and characterized for size distribution using low-angle laser light scattering (LALLS) and SEM. The particles were predominantly less than 2 μm in diameter, with an average size of 1 μm ± 0.5 μm. During the test, 14 mg was placed beneath each pin to simulate conditions where bone cement debris is present (Figure 5).

Surface topography measurements and wear rate calculations were performed as described above. In addition, Inductively Coupled Plasma Optical Emission Spectrometry (ICP-OES) was performed to determine any metals released into the lubricant (see Section 2.5.4).

#### 2.4.3. Determining Wear Rates

For the standard wear test without PMMA bone cement (referred to as the “standard wear test”), wear rates were determined by subjecting the coated and uncoated substrates to a total of 1 million cycles, with mass loss measurements taken at every 250,000 cycles. This method allows for the calculation of a wear rate using linear regression as outlined in ASTM standards 2025 and F732. The linear wear rate is advantageous as it provides a consistent measure of wear over time, reducing the influence of any early-stage irregularities in the wear process.

For the wear test with PMMA bone cement particles (referred to as the “PMMA test”), the wear rates were established from two data points: the mass of the pins immediately before and after the test. This test was performed over 500,000 cycles; however, wear rates are reported normalized for 1 million cycles for ease of comparison. No apparent embedding of PMMA particles on the surfaces of the pins was observed after cleaning, which suggests that the reported wear rates correctly reflect worn-off polyethylene without confounding embedding of bone cement.

### 2.5. Imaging and Spectroscopy

#### 2.5.1. Light Microscopy

Surface morphology alterations of pins and metal discs were qualitatively assessed using a ZEISS Stemi 2000-C stereomicroscope (Oberkochen, Germany), which provides distortion-free images. With 0.4× and neutral lenses, and either single or dual lateral illumination, the instrument was used to identify morphological changes on the surfaces.

#### 2.5.2. Scanning Electron Microscopy (SEM)

Fine-scale morphological and compositional analysis was conducted using a Jeol Field Emission Scanning Electron Microscope (IT500HR, JEOL, Tokyo, Japan), which was equipped with an Energy Dispersive X-ray Spectroscopy (EDS) detector (Ultim Max, Oxford, UK) and an Electron Backscatter Diffraction (EBSD) detector (C-nano, Oxford Inc., Oxford, UK). Surface features both prior to and following the standard wear tests, as well as the wear tests that included PMMA bone cement particles were recorded. An accelerating voltage of 15.0 kV and working distances noted in the image metadata for each sample were chosen. Comprehensive SEM scans and further details are included in the supplement for extended review.

#### 2.5.3. Raman Spectroscopy

Before and after the standard wear test, one disc from each group was scanned on a Horiba LabRAM HR Evolution Raman spectrometer (Irvine, CA, USA). Using a 532 nm laser, an area inside and outside the wear scar for each disc was excited, and the Raman emission spectra were captured and analyzed using Horiba LabSpec 6 software. For each scan, a fraction of the excitation light was diverted to a reference sample of ortho-Xylene, and emission overlaid on the spectrometer. These sharp Raman peaks at 540, 750, 830, 900, and 1000 cm^−1^ were used to check for any potential instrumental drift.

#### 2.5.4. Inductively Coupled Plasma Optical Emission Spectrometry (ICP-OES)

After completion of the PMMA wear test, deionized water was added to each wear chamber and brought back to the exact starting weight to account for any evaporation of the lubricant during testing. After thorough mixing, 2 mL aliquots from each wear chamber were pipetted into Eppendorf^®^ containers and shipped to an Analytical Chemistry laboratory. Pressurized digestion by means of nitric and hydrofluoric acid using a specific time-heat-pressure microwave protocol was applied to assure the complete decay of any metal particles, including oxides [42]. As described in the aforementioned study, using ICP-OES, such a protocol allows the simultaneous detection of several metal elements in the range of 0.05 parts per million (ppm). Here, we analyzed four elements: titanium (Ti), cobalt (Co), chromium (Cr), and molybdenum (Mo).

### 2.6. Statistics

To investigate the relationship between the wear rate and the two independent variables, substrate type and coating technique, as well as their potential interaction, a two-way Analysis of Variance (ANOVA) using OriginPro software (Version 2022b, Build No. 9.950167) was conducted. The alpha level was set at 0.05 for all statistical tests.

Prior to the ANOVA, the data were checked for normality and homoscedasticity to ensure compliance with the test’s assumptions. Following the ANOVA, a Bonferroni-adjusted post hoc analysis was performed to control for the inflation of Type I error rate due to multiple comparisons.

To analyze any increase in UHMWPE wear rate due to the addition of bone cement in the lubricant, a series of paired *t*-tests were conducted for each sample type. Each *t*-test was two-tailed with an alpha level set at 0.05.

## 3. Results

### 3.1. Pin-on-Disc Wear Test Using Bovine Serum

#### 3.1.1. Polyethylene Wear

In the evaluation of polyethylene wear, the pin-on-disc wear test using bovine serum was employed. The average wear rate (WR) of polyethylene, expressed in milligrams per million cycles (mg/MC), served as the primary metric for assessing wear.

The ANOVA results indicated significant differences across the factors of substrate type and coating technique. Specifically, the substrate type (CoCrMo vs. Ti6Al4V) showed a significant effect on wear rate with *p* = 0.003, indicating that the type of substrate material substantially impacts wear performance. Similarly, the coating technique also significantly affected wear rate (*p* < 0.001). The interaction between the substrate and coating was also found to be significant, although borderline (*p* = 0.049), suggesting that the effect of the coating technique on wear rate depends on the type of substrate used.

The test results revealed distinct performances among the different substrate and coating combinations. For the CoCrMo substrate, the uncoated samples showed a moderate wear rate, which was significantly reduced by the Arc-PT coating, demonstrating its efficacy in enhancing wear resistance. The HiPIMS coating led to an increase in wear rate, which was strongly attenuated when post-treatment was applied, indicating the post-treatment’s role in improving the coating’s protective qualities.

Ti6Al4V substrates showed higher baseline wear rates for uncoated samples when compared to CoCrMo. However, similar to CoCrMo, the application of Arc-PT and HiPIMS-PT coatings reduced wear rates, confirming the beneficial effects of these coatings across different substrates. The HiPIMS coating without post-treatment also resulted in increased wear rates for Ti6Al4V, mirroring the trend observed with CoCrMo.

The standard deviation (SD) associated with the average wear rates indicated the variability within the test results, which was observed across all samples. Notably, the HiPIMS-PT coating on CoCrMo showed higher variability, as reflected by its SD value.

**Figure 6.**
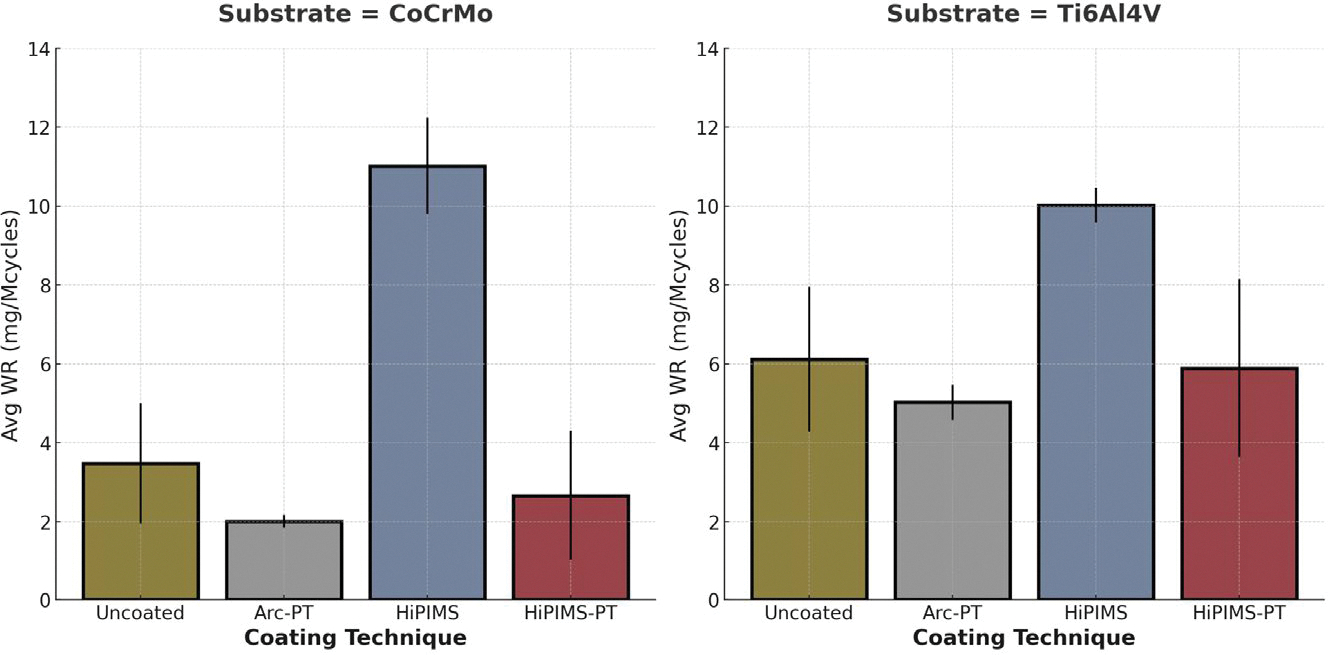
Average wear rates for standard wear test for CoCrMo and Ti6Al4V substrates. The coatings tested include arc physical vapor deposition with post-treatment (Arc-PT), high-power impulse magnetron sputtering (HiPIMS), and HiPIMS with post-treatment (HiPIMS-PT). The error bars represent the standard deviation (SD).

The data presented in Figure 6, along with the findings from the statistical analysis, clearly indicate the substantial influence of both substrate type and coating technique on the wear resistance of the materials.

#### 3.1.2. Surface Appearance and Roughness

**Figure 7.**
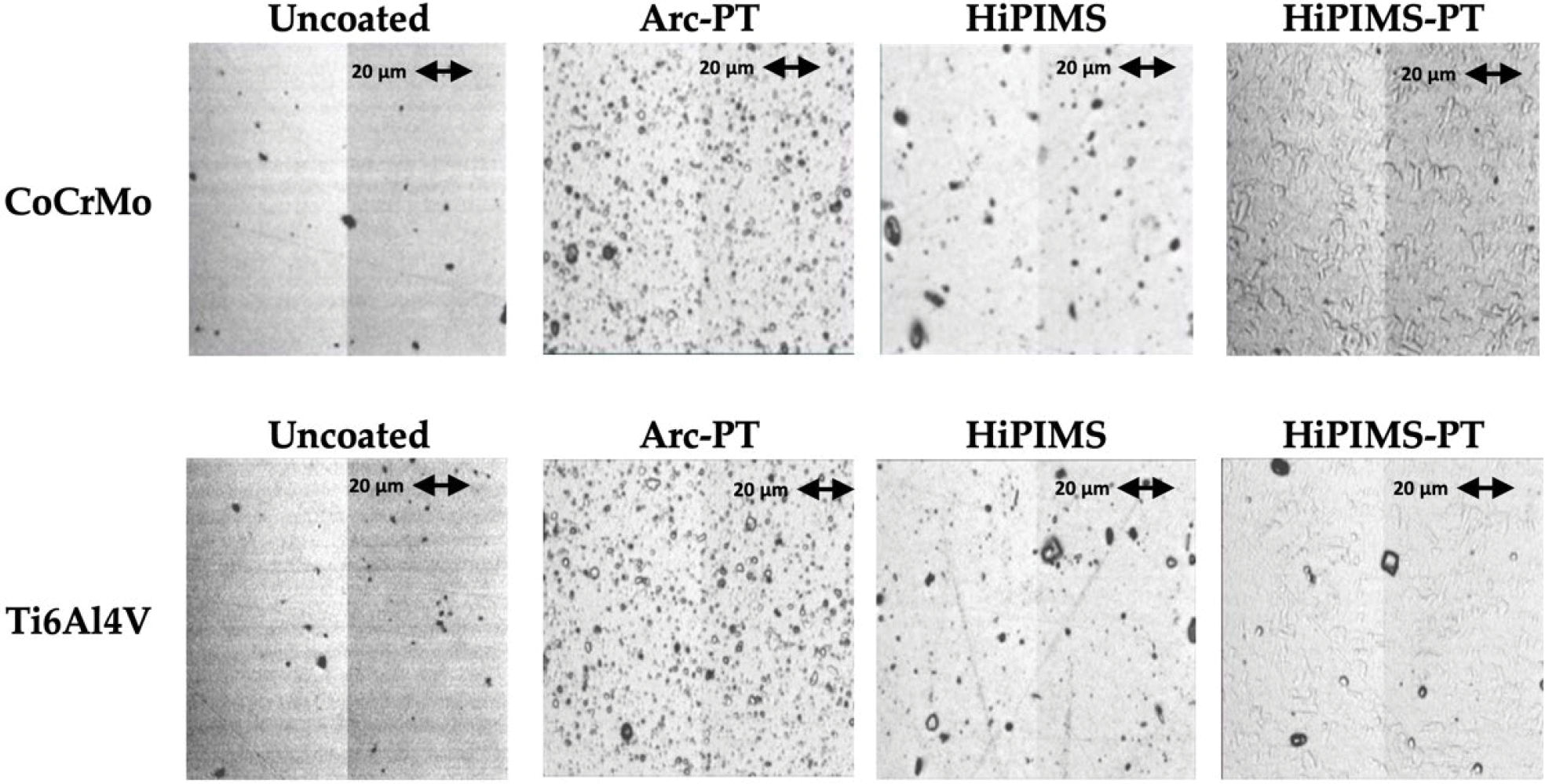
Comparative surface appearances of CoCrMo and Ti6Al4V substrates (uncoated and coated) before the wear test. The coatings illustrated include arc physical vapor deposition with post-treatment (Arc-PT), high-power impulse magnetron sputtering (HiPIMS), and HiPIMS with post-treatment (HiPIMS-PT).

The surface morphology of the CoCrMo and Ti6Al4V alloy discs, as observed through light microscopy, are shown in Figure 7. Roughness values obtained through white light interferometry before and after the pin-on-disc (POD) wear test are displayed in Table 3.

**Figure 8.**
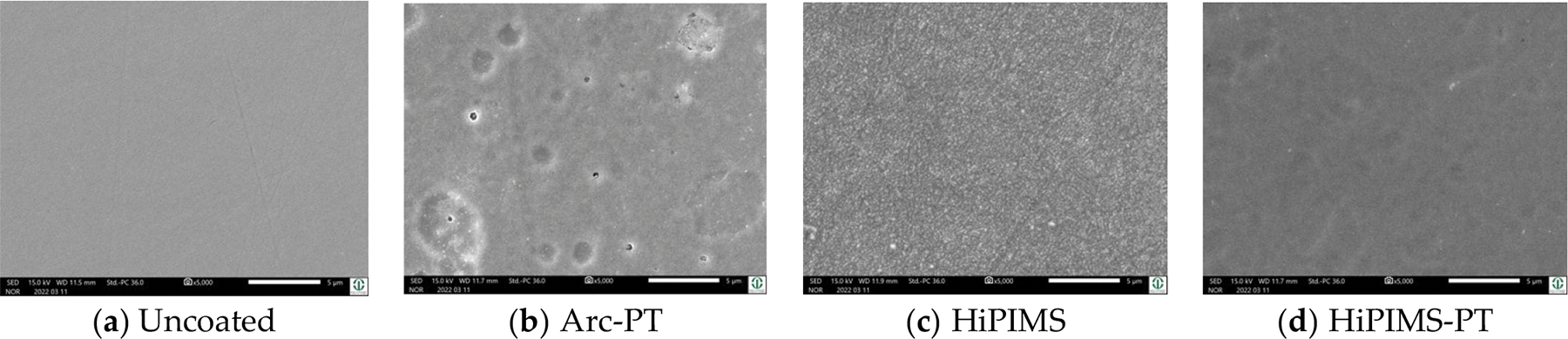
SEM images of CoCrMo substrate discs; uncoated, Arc-PT, HiPIMS, and HiPIMS-PT after wear testing. The uncoated CoCrMo surfaces showed traces of wear, the Arc-PT retained its coating morphology, and the HiPIMS surfaces revealed increased irregularities. In contrast, the HiPIMS-PT surfaces showed minimal morphological changes, consistent with a less pronounced roughness variation.

The initial examination of uncoated CoCrMo and Ti6Al4V substrates showed minimal surface imperfections, but post-wear testing revealed wear tracks. In both cases, the increases in roughness were not significant, although an increase of 7.948 nm for Ti6Al4V underscores the higher wear susceptibility of titanium. The arc physical vapor deposition with post-treatment (Arc-PT) process on CoCrMo, characterized by voids and droplet-induced speckle patterns, initially registered a high arithmetic roughness value of approximately 30 nm. After wear, a roughness reduction of 7.27 nm indicated a trending smoothing of the surface, in contrast to Ti6Al4V, which stayed unaffected. Only the HiPIMS coatings with post-treatment (HiPIMS-PT) showed significant changes in roughness. This is mainly an effect of their tight standard deviations before and after wear. Their densely textured pattern with minor defects after wear resulted in a minor roughness decrease of 2.30 nm for CoCrMo, while Ti6Al4V experienced an increase of 4.57 nm, reflecting the varied impact of the abrasive challenge on different coatings and substrates. The SEM analysis (provided for all conditions in the Supplementary Materials) further supported these observations (Figure 8).

#### 3.1.3. Raman Spectroscopy of Discs

For the uncoated Ti6Al4V and CoCrMo discs, the Raman spectra before and after wear testing showed no significant shifts, suggesting that the surfaces maintained their molecular composition without detectable alterations by the Raman spectroscopy, i.e., in particular, none of the coatings developed a polyethylene transfer film.

**Figure 9.**
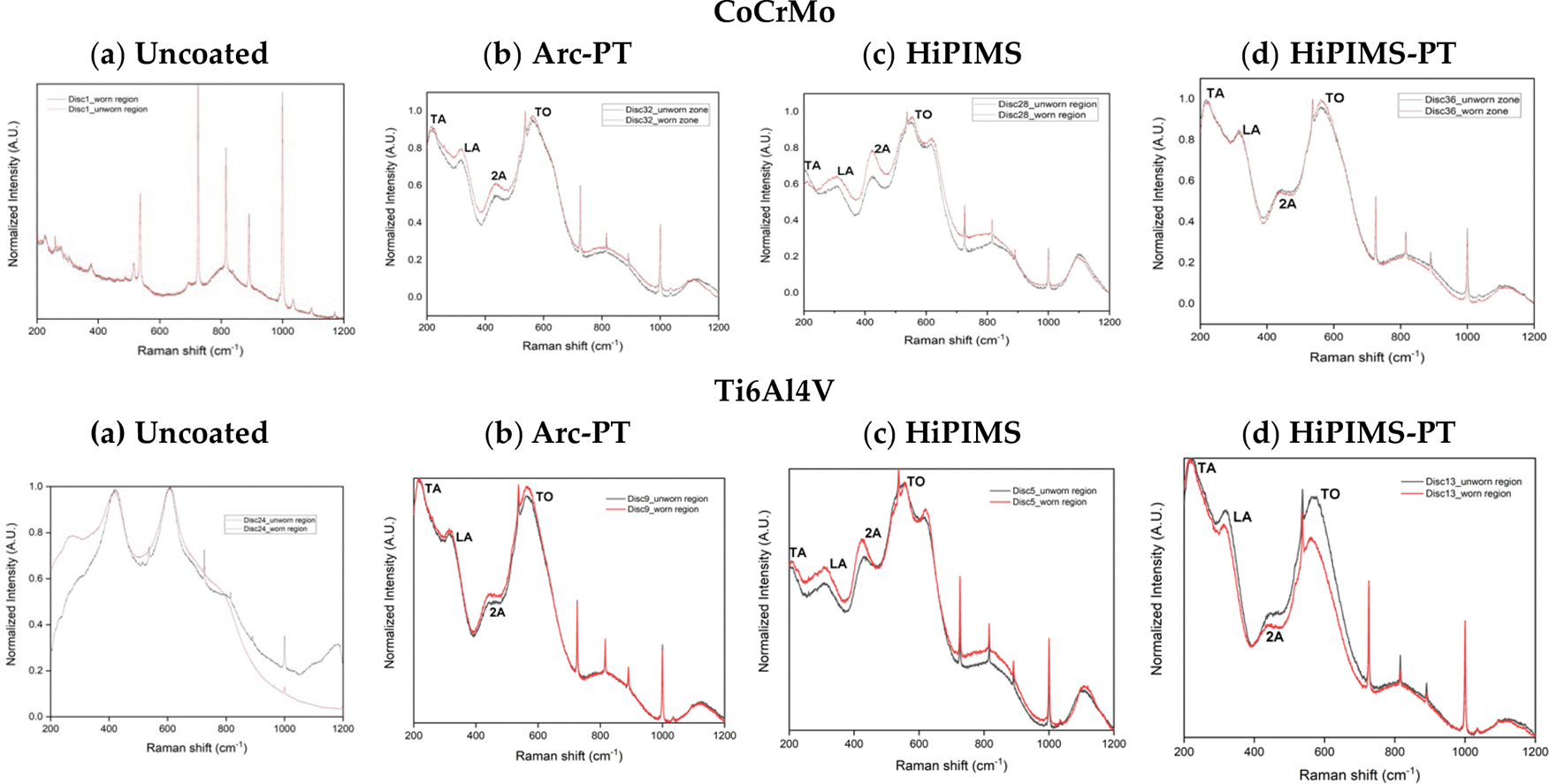
Comparative Raman spectra of CoCrMo and Ti6Al4V substrates with various coatings: (**a**) uncoated substrates, (**b**) substrates with post-treated Arc (Arc-PT) coatings, (**c**) substrates with high-power impulse magnetron sputtering (HiPIMS) coatings, and (**d**) substrates with post-treated HiPIMS (HiPIMS-PT) coatings. For each substrate and treatment type, the black line represents the Raman spectrum from the unworn zone, while the red line indicates the spectrum from the worn region (wear zone), providing insight into the structural and compositional changes induced by wear.

For TiN coated discs, four peaks at 235, 320, 440, and 570 cm^−1^ were observed. These peaks correspond to transverse acoustic (TA), longitudinal acoustic (LA), second-order acoustic (2A), and transverse optical (TO) modes of TiN [43]. Two unidentified peaks were observed at 800 and 1100 cm^−1^. Another peak in HiPIMS and shoulder in Arc-PT at 617 cm^−1^ were observed, but also could not be identified. In the wear track, minor shifts were observed in the 2A peak of the HiPIMS (blue-shifted 8.4 cm^−1^) and HiPIMS-PT (blue-shifted 4.9 cm^−1^), but only for coatings on CoCrMo substrates. The wavenumber of Raman peaks can be influenced by residual stresses present in the coating, and if the magnitude of these stresses follows a gradient across coating depth, surface damage to the coating could expose a coating layer closer the coating/substrate interface [43–45]. However, only minor shifts were observed in the spectra of coated discs, hinting at microstructural adjustments attributable to the mechanical stress experienced during the wear test rather than worn off material (Figure 9a,b). Notably, the HiPIMS-PT coatings demonstrated no Raman shifts at all (Figure 9c).

### 3.2. PMMA Bone Cement Particles Wear Test Results

#### 3.2.1. Polyethylene Wear

**Figure 10.**
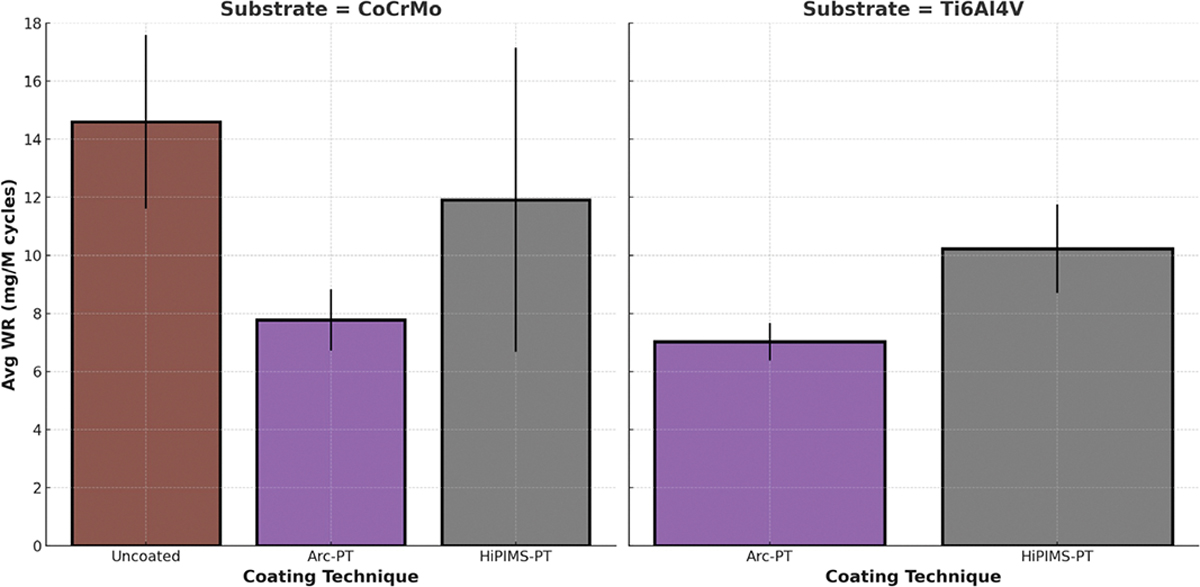
Polyethylene average wear rate after PMMA bone cement particle challenge for CoCrMo and Ti6Al4V substrates. The coatings tested include arc physical vapor deposition with post-treatment (Arc-PT), high-power impulse magnetron sputtering (HiPIMS), and HiPIMS with post-treatment (HiPIMS-PT). The error bars represent the standard deviation (SD).

Polyethylene wear was highest for the uncoated CoCrMo disc (14.6 mg/MC) and lowest for Arc-PT (7.7 and 7.0 mg/MC for the CoCrMo and TiAlV substrates, respectively) (Figure 10).

For the uncoated CoCrMo specimens, the wear rate significantly increased post PMMA application (*p* = 0.049), indicating a pronounced vulnerability to abrasive wear. Despite a significant increase for the Arc-PT coated CoCrMo specimens (to 7.77 mg/MC, *p* = 0.014), the wear rate was substantially lower than that of the uncoated specimens, demonstrating the coating’s relative protective performance. Although the HiPIMS-PT coating on CoCrMo showed an increased wear rate to 11.91 mg/MC, this change was not statistically significant (*p* = 0.115).

For the Ti6Al4V substrate, the HiPIMS-PT coated discs exhibited a significant increase in wear rate to 10.22 mg/MC (*p* = 0.018) following PMMA application. Despite this significant increase, the HiPIMS-PT coating wear rate remained lower than that of the uncoated CoCrMo, suggesting that it provides a considerable protective effect. In contrast, the Arc-PT coating on Ti6Al4V demonstrated wear resistance, with no significant increase in wear rate observed (7.02 mg/MC, *p* = 0.108), underscoring the effectiveness of the Arc-PT coating in protecting against the abrasive action of PMMA particles, particularly when applied on titanium surfaces. The observations are summarized in Table 4.

#### 3.2.2. Metal Release into Bovine Serum

In the uncoated state, CoCrMo revealed substantial metal release, with the detected average concentrations of cobalt (Co) at 5.947 ± 1.862 ppm, chromium (Cr) at 2.563 ± 0.766 ppm, and molybdenum (Mo) at 0.527 ± 0.174 ppm. These figures represent the inherent propensity of the substrate material to release metal ions under wear conditions, establishing a baseline for comparing the efficacy of various coatings.

Upon the application of arc physical vapor deposition with post-treatment (Arc-PT) and high-power impulse magnetron sputtering with post-treatment (HiPIMS-PT) coatings, a dramatic decline in metal ion concentrations was observed. Specifically, the Arc-PT technique reduced the cobalt levels in CoCrMo substrates below the detection limit, manifesting a reduction of at least 99% compared to the uncoated substrate. This underscores the coating’s role as a formidable barrier against ion leakage.

Similarly, the HiPIMS-PT method showed a significant decrease in cobalt release, maintaining the ion concentration below the detection limit. This consistent cobalt containment across both coating technologies reinforces their validity as effective measures to mitigate metal ion release.

Both substrates, either coated with Arc-PT or HiPIMS-PT, presented with identical titanium (Ti) concentrations (Table 5). Although uncoated Ti6Al4V substrates were not tested with bone cement in this investigation, the coated samples exhibited a commendable restriction of titanium release, which is likely reflective of additional polishing of the microdroplets. In aggregate, the obtained values are lower than 1/3 of the total metal release from uncoated CoCrMo.

#### 3.2.3. Wear Appearances and Morphology

**Figure 11.**
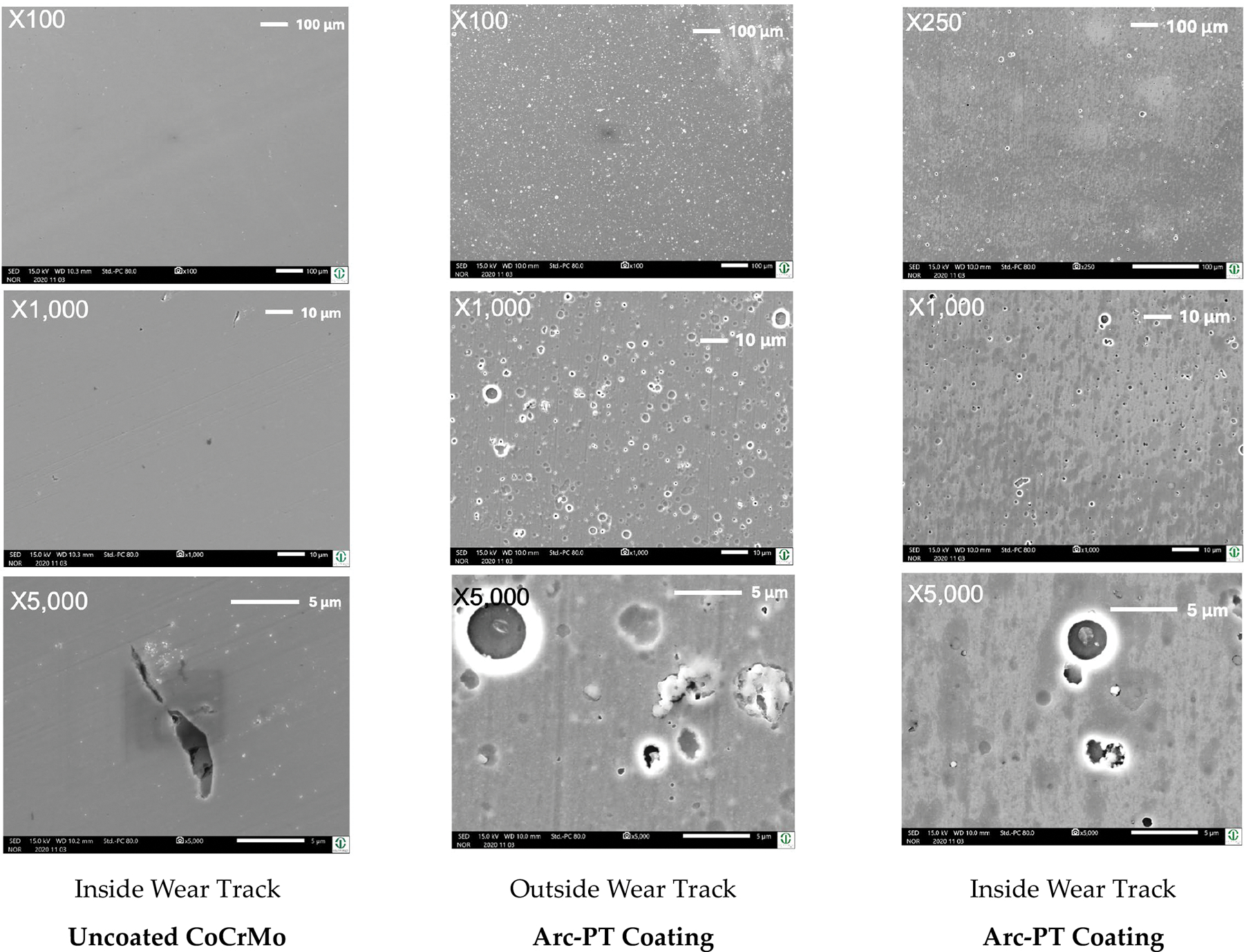
Scanning electron microscope (SEM) images at multiple magnifications (×100, ×250, ×1000, and ×5000) showcasing wear track morphologies of Arc-PT coated CoCrMo substrates against uncoated control.

The SEM micrographs at various magnifications (Figure 11) provide a comprehensive overview of the surface topography and wear patterns. For the uncoated CoCrMo, the images at lower magnifications (×100, ×250) reveal a relatively uniform surface with minor pre-existing imperfections. As we observe at higher magnifications (×1000, ×5000), these imperfections become more pronounced, with wear tracks visibly aligned with the direction of abrasive action, suggesting a plowing effect during the wear test.

On the other hand, the Arc-PT coated CoCrMo surfaces demonstrate a distinct morphology. Even at lower magnifications, the coating’s granular structure is evident, attributed to the deposition process. Interestingly, the images inside the wear track show fewer and less severe defects than those outside the wear track, implying that the wear process may have a smoothing effect on the roughness within the wear track. This could be due to the removal of asperities or the redistribution of material during the testing.

In relation to roughness and smoothness, these visual assessments correlate with the quantitative roughness data. The reduction in roughness inside the wear track as opposed to the outside could indicate material compaction or a wear-induced polishing effect. This is in line with the negative average change values observed for the Arc-PT on Ti6Al4V and HiPIMS-PT on Ti6Al4V coatings post testing, which signify a smoother surface post wear.

The SEM analysis thus confirms the protective role of the coatings, with the Arc-PT and HiPIMS-PT coatings showing an ability to maintain surface integrity under wear conditions, as evidenced by the less significant morphological changes within the wear tracks. The durability of these coatings is paramount in biomedical applications where wear resistance is critical for implant longevity. The findings suggest that while the coating techniques improve the surface characteristics against abrasive wear, there remains a necessity to further optimize the coatings to reduce the occurrence of defects that can compromise wear resistance.

#### 3.2.4. Roughness Changes after PMMA Particle Challenge

On average, the roughness of the uncoated CoCrMo discs increased, while roughness decreased for all coated discs, corroborating the observations made using SEM. In a way, the PMMA particles served as a polishing slurry for the TiN coatings. This finding, however, was only significant for the HiPIMS-PT coating, as can be seen in Table 6.

## 4. Discussion

The overarching goal of this study was to scrutinize the effectiveness of titanium nitride (TiN) coatings, applied via advanced techniques on CoCrMo and Ti6Al4V substrates. Our findings corroborate the notion that such coatings serve as a substantial barrier against wear and metal ion release, potentially ameliorating the longevity of orthopedic implants. Notably, coatings applied through arc physical vapor deposition with post-treatment (Arc-PT) and high-power impulse magnetron sputtering with post-treatment (HiPIMS-PT) exhibited significant prowess in curtailing wear.

The wear tests, standardized and augmented with polymeric PMMA particles, delineate the performance matrix of the uncoated and coated substrates under simulated physiological conditions. In the abrasive milieu of PMMA particles, the coatings—Arc-PT and HiPIMS-PT—demonstrated a notable reduction in wear rates for both CoCrMo and Ti6Al4V substrates. Particularly, the Arc-PT coating on CoCrMo underscored its superior wear resistance by halving the wear rate compared to the uncoated specimens, indicating a significant enhancement in durability. Even though the HiPIMS-PT coatings exhibited elevated wear rates in comparison, they still manifested a considerable protective effect, underscoring the coatings’ efficacy. The observed differences may be attributed to the occurrence of micro-depressions, which are higher in number on the Arc-PT surface. While considered a coating artifact, these depressions entrap third-body particles and serve lubricant reservoirs. This study also made clear that microdroplets, even low in number, cannot be left on the surface and have to be removed during post-treatment. Polyethylene wear was two to four times higher for the HiPIMS coating when post-treatment was skipped.

The critical examination of metal ion release in bovine serum lubricant due to third-body particles offers insights into the biocompatibility and safety of orthopedic implants. Employing Inductively Coupled Plasma Optical Emission Spectrometry (ICP-OES), our investigation quantified metal ion concentrations, an endeavor useful in assessing the potential health implications post implantation. For the uncoated CoCrMo substrates, the data revealed a notable baseline ion discharge, providing a comparative benchmark to gauge the efficacy of the coatings. Remarkably, the application of Arc-PT and HiPIMS-PT coatings precipitated a stark reduction in metal ion release. Cobalt levels in CoCrMo substrates plummeted below detection limit, a decrease of at least 99%, exemplifying the coatings’ capacity as formidable barriers against ion leaching. These findings illuminate the pronounced effect of coatings in enhancing the safety profile of implants by minimizing ion release, which could translate into a reduced risk of systemic distribution and organ accumulation, thereby fortifying the long-term compatibility of orthopedic devices.

The surface morphology of orthopedic implant materials is a pivotal factor in their performance and longevity. While uncoated discs maintained their molecular integrity, the coated discs exhibited minor Raman shifts indicative of microstructural adaptations due to shear. This effect might be expected since only little material is removed from the coated surfaces. In contrast, uncoated CoCrMo disks, at higher magnifications (×1000 and ×5000), show wear tracks visibly aligned with the direction of abrasive action, suggesting material removal due to a microplowing effect. In comparison, the Arc-PT coated CoCrMo surfaces present a distinct morphology; even at lower magnifications, the coating’s granular structure, attributed to the deposition process, is evident. Notably, the SEM images inside the wear track show fewer and less severe defects than those outside the wear track, implying that the wear process may have a smoothing effect on the roughness within the wear track, possibly due to the removal of asperities or the redistribution of material during testing. Correlating these visual assessments with quantitative roughness data, the reduction in roughness inside the wear track as opposed to the outside suggests material compaction or a wear-induced polishing effect. This is consistent with the negative average change values observed for the Arc-PT on Ti6Al4V and HiPIMS-PT on Ti6Al4V coatings post testing.

In the context of the existing literature, particularly the study conducted by our group on DLC coatings, the current research presents a significant advancement in coating stability and delamination resistance. Our previous study, focused on DLC coatings, highlighted issues of delamination under certain testing conditions, raising concerns about their practical applicability [34]. However, in our current study, we observed a high coating integrity. Notably, the TiN coatings applied via arc evaporation (Arc-PT) and high-power impulse magnetron sputtering with post-treatment (HiPIMS-PT) demonstrated exceptional stability without any delamination, even under the challenging conditions of wear testing with PMMA bone cement particles. This positive outcome signals that recent advancements in deposition techniques and post-treatment processes have effectively addressed the delamination issues noted in the past. Further investigations, particularly under higher contact pressures and more complex motion patterns akin to those in knee or hip simulators, are essential to fully ascertain the long-term performance and reliability of these coatings in clinical scenarios.

Upon reviewing the wear testing results and the subsequent analysis, it is crucial to address the inherent limitations of this study while simultaneously highlighting its strengths. Pin-on-disc testing, while not a perfect representation of the complex biomechanical environment of joint replacements, offers a controlled, repeatable, and standardized method for evaluating the tribological performance of orthopedic implant materials. Although actual joint movements are more intricate, the testing conducted provides valuable insights into the wear characteristics and potential longevity of the materials tested. The use of “flat” discs, rather than complex shaped implant components, allowed for a focused examination of the coatings’ effectiveness. While this approach does not capture the full geometrical complexity of implant surfaces, it does ensure that the results are directly attributable to the material and coating properties, without other confounding influences. The study’s cycle numbers, capped at 1 million for the standard wear test and 0.5 million for the PMMA test, fall short of the 5 million cycles that are required for hip and knee wear testing following the respective ISO standards. However, these cycle numbers were deemed sufficient to reveal significant trends and differences between the coatings and substrates. It is also worth noting that the inclusion of PMMA particles, in the absence of approved standards for third-body wear [46,47], was executed with a commitment to realism, utilizing particles derived from polymerized PMMA to reflect the clinical scenario more accurately. The introduction of PMMA particles, while a deviation from natural joint conditions, was thoughtfully executed to mimic the abrasive environment within a joint post implantation. This aspect of our study provides a more challenging and therefore more telling assessment of the materials’ performance.

From an industrial perspective, our study encourages the ongoing refinement of coating techniques to harness the full potential of TiN’s tribological benefits. The consequential reduction in wear debris holds promise for diminishing the incidence of osteolysis and aseptic loosening (primary causes of joint replacement failure), thus potentially prolonging the functional lifetime of the implants. Future studies should also pay more attention to the microstructural properties of the substrate and coating since those features ultimately define the longevity of the implant.

## 5. Conclusions

The tribological investigation of titanium nitride (TiN) coatings, particularly those applied via arc evaporation and high-power impulse magnetron sputtering followed by post-treatment, revealed a pronounced reduction in wear, ranging from 20 to 40% depending on the substrate composition. It became evident that TiN coatings require post-treatment. Leaving HiPIMS coatings untreated causes high polyethylene wear rates. Under adverse conditions, i.e., the introduction of bone cement particles, the coatings not only strongly decreased the release of potentially harmful ions such as cobalt, chromium, and molybdenum, but they also kept polyethylene wear contained. In contrast, for uncoated CoCrMo samples, polyethylene wear increased four-fold. The implications are profound, especially in the realm of CoCrMo-based implants, which have historically raised concerns over adverse tissue reactions.

## Supplementary Material

Supplementary material

**Supplementary Materials:** The following supporting information can be downloaded at: https://www.mdpi.com/article/10.3390/lubricants12030096/s1, Figure S1; Scanning Electron Microscope Observations of CoCrMo Alloy Discs; Figure S2: SEM Images of Arc-PT Coated CoCrMo Substrate; Figure S3: SEM Images of HiPIMS-PT Coated CoCrMo Substrate; Figure S4: SEM Images of Arc-PT Coated TiAlV Substrate; Figure S5: SEM Images of HiPIMS-PT Coated TiAlV Substrate; Table S1: Configuration of PIN ON DISC (POD) Test with Bone Cement. Figure S6: Light Microscopy Images of Polyethylene Pins (pins #1–35); Figure S7: Scanning Optical Microscopy of Polyethylene Pins After Wear Testing; Figure S8: Scanning Optical Microscopy of Polyethylene Pins After Wear Testing; Figure S9: Scanning Optical Microscopy of Polyethylene Pins After Wear Testing; Figure S10: Scanning Optical Microscopy of Polyethylene Pins After Wear Testing; Figure S11: Scanning Optical Microscopy of Polyethylene Pins After Wear Testing; Figure S12: Scanning Optical Microscopy of Polyethylene Pins After Wear Testing.

## Figures and Tables

**Table 1.** Arithmetic mean roughness values (R_a_) in nanometess Onm) for uoated and uncoated discs as received for wear testing.

Substrate	Coating Technique	R_a_ ± SD (nm)

CoCrMo	Uncoated	4.16 ± 0.35
	Arc-PT ^1^	35.24 ± 7.35
	HiPIMS ^2^	5.36 ± 3.92
	HiPIMS-PT ^3^	14.92 ± 1.28

Ti6Al4V	Uncoated	17.23 ± 3.64
	Arc-PT	42.50 ± 4.33
	HiPIMS	30.40 ± 5.76
	HiPIMS-PT	27.95 ± 2.83

1 Arc-PT: Arc deposition with post-treatment to enhance surface characteristics.

2 HiPIMS: High-power impulse magnetron sputtering without post-treatment, providing the coating in its as-deposited state.

3 HiPIMS-PT: High-power impulse magnetron sputtering followed by post-treatment to enhance surface characteristics. All values of R_a_ are given in nanometers (mm) as mean ± standard deviation.

The table illustrates the R_a_ values for each substrate with and without coatings applied. Roughness values obtained by Rush investigators following the description in the Section 2.4.1.

**Table 2.** Comparison of coating thickness and hardness for Arc PT, HiPIMS, and HiPMS-PT.

Coating	Thickness (μm)	Hardness ± SD (GPa)

Arc-PT	4	30.3 ± 1.3
HiPIMS	5	33.5 ± 1.8
HiPIMS-PT	5	32 ± 0.8

The table summarizes the measured thickness and hardness values for titanium nitride (TiN) coatings applied using arc evaporation (Arc PT) and high-power impulse magnetron sputtering (HiPIMS), with and without post-treatment (PT).

**Table 3.** Arithmetic mean roughness (Ra) with the corresponding standard deviation (SD) measured in nanometers (nm) before (Pre) and after (Post) the wear test.

Coating	Substrate	R_a_ ± SD (nm) Pre	R_a_ ± SD (nm) Post	*p* Value

Uncoated	CoCrMo	4.16 ± 0.58	4.70 ± 0.29	0.309
	Ti6Al4V	17.23 ± 0.20	25.18 ± 8.56	0.256

Arc-PT	CoCrMo	35.24 ± 7.24	27.97 ± 3.91	0.073
	Ti6Al4V	42.50 ± 4.59	43.75 ± 8.52	0.637

HiPIMS	CoCrMo	15.36 ± 0.80	17.94 ± 0.96	0.124
	Ti6Al4V	30.40 ± 0.91	34.41 ± 6.27	0.421

HiPIMS-PT	CoCrMo	14.92 ± 1.38	12.61 ± 0.92	**0.044**
	Ti6Al4V	27.95 ± 5.50	35.52 ± 5.61	**0.046**

*p* values with a value below 0.05 (in bold) denote a statistically significant change from Pre to Post.

**Table 4.** Wear rate (WR, mg/MC) of CoCrMo and Ti6Al4V substrates, uncoated and coated with Arc-PT and HiPIMS-PT, before and after the PMMA bone cement particle wear test.

Coating	Substrate	WR ± SD (mg/MC) Pre	WR ± SD (mg/MC) Post	*p* Value

Uncoated	CoCrMo	3.47 ± 1.51	14.6 ± 2.99	**0.049**

Arc-PT	CoCrMo	2.0 ± 0.16	7.77 ± 1.05	**0.014**
	Ti6Al4V	5.02 ± 0.44	7.02 ± 0.65	0.108

HiPIMS-PT	CoCrMo	2.65 ± 1.64	11.91 ± 5.23	0.115
	Ti6Al4V	5.88 ± 2.25	10.22 ±1.52	**0.018**

Values are presented as mean ± standard deviation. *p*-values in bold indicate significance.

**Table 5.** Content of metal in bovine serum lubricant measured using ICP-OES post bone cement particle wear test.

Coating	Substrate	Co	Mo	Cr	Ti

Uncoated	CoCrMo	5.947 ± 1.862	0.527 ± 0.174	2.563 ± 0.766	ND

Arc-PT	CoCrMo	ND	ND	ND	2.733 ± 1.124
	Ti6Al4V	ND	ND	ND	2.133 ± 1.106

HiPIMS-PT	CoCrMo	ND	ND	ND	3.333 ± 3.784
	Ti6Al4V	ND	ND	ND	2.266 ± 2.532

Values for each element are expressed in ppm. Values below the detection limits are reported as ‘ND’ (not detected). Detection limits for the elements are as follows: Ti > 0.16 ppm, Co > 0.08 ppm, Mo > 0.03 ppm, and Cr > 0.08 ppm.

**Table 6.** Average surface roughness (Ra, nm) of CoCrMo and Ti6Al4V substrates, uncoated and coated with Arc-PT and HiPIMS-PT, before and after the PMMA bone cement particle wear test.

Coating	Substrate	R_a_ ± SD (nm) Pre	R_a_ ± SD (nm) Post	*p* Value

Uncoated	CoCrMo	4.707 ± 1.335	5.652 ± 0.600	0.295

Arc-PT	CoCrMo	27.967 ± 1.3.998	22.445 ± 2.834	0.087
	Ti6Al4V	43.748 ± 17.387	31.473 ± 4.715	0.120

HiPIMS-PT	CoCrMo	12.613 ± 3.550	9.991 ± 0.700	0.053
	Ti6Al4V	32.524 ± 3.647	21.346 ± 5.365	0.031

Values are presented as mean ± standard deviation. *p*-values in bold indicate significance.

## Data Availability

The datasets generated and/or analyzed during the current study are available from the corresponding author, Markus Wimmer, on reasonable request. Additional data that support the findings of this study are included within the article and its Supplementary Materials.
