# Supplementary material for: Titanium Nitride Coatings on CoCrMo and Ti6Al4V Alloys: Effects on Wear and Ion Release
Source: Lubricants. Author manuscript; Available in PMC 2026 Feb 25. (PMC12931815; doi:10.3390/lubricants12030096)
Supplement: Supplementary material [file NIHMS2141616-supplement-Supplementary_material.pptx]

## Slide 1
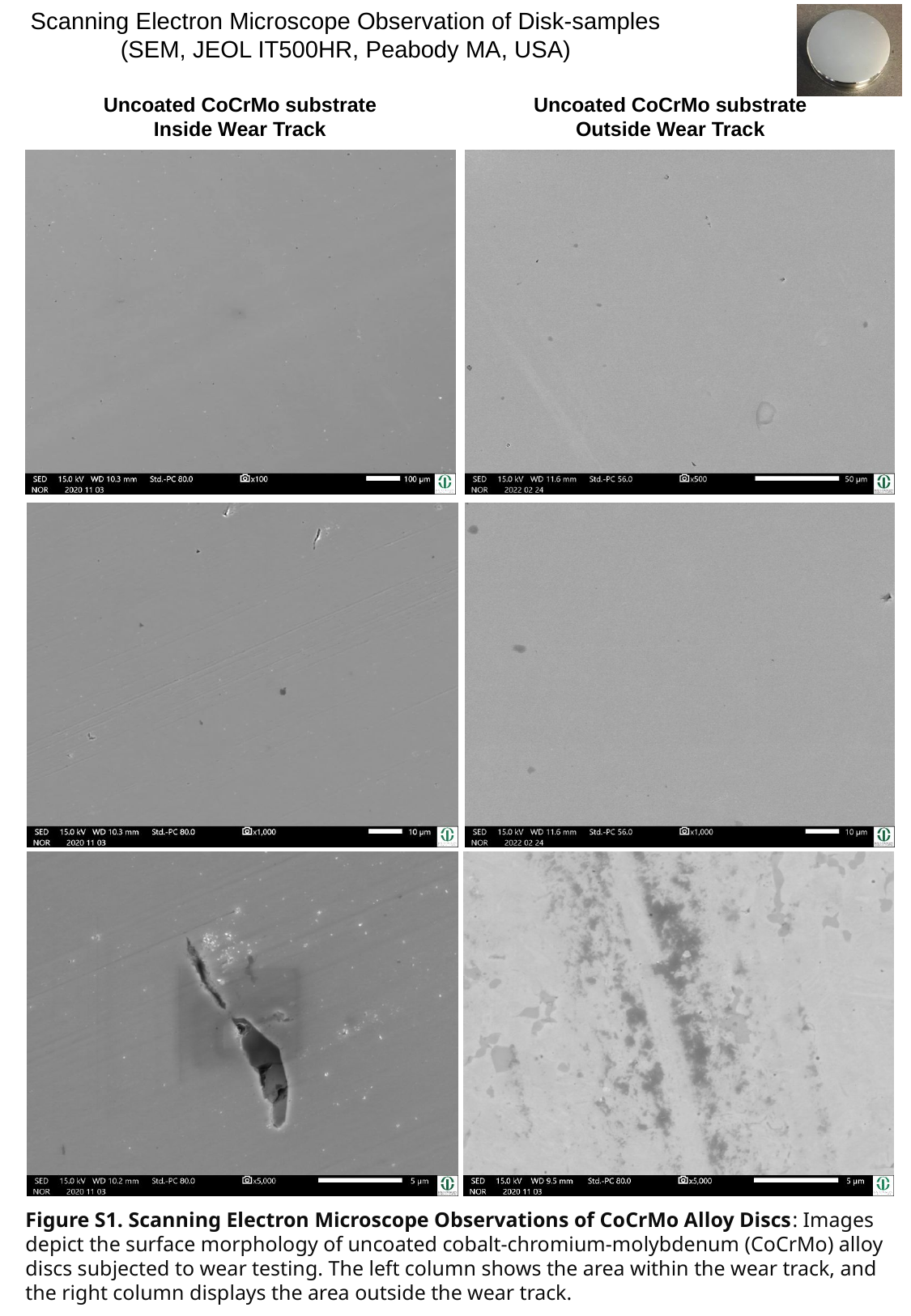

Scanning Electron Microscope Observation of Disk-samples
(SEM, JEOL IT500HR, Peabody MA, USA)
Uncoated CoCrMo substrate
Inside Wear Track
Uncoated CoCrMo substrate
Outside Wear Track
X100
X100
X1,000
X1,000
X5,000
X5,000
Figure S1. Scanning Electron Microscope Observations of CoCrMo Alloy Discs: Images depict the surface morphology of uncoated cobalt-chromium-molybdenum (CoCrMo) alloy discs subjected to wear testing. The left column shows the area within the wear track, and the right column displays the area outside the wear track.

## Slide 2
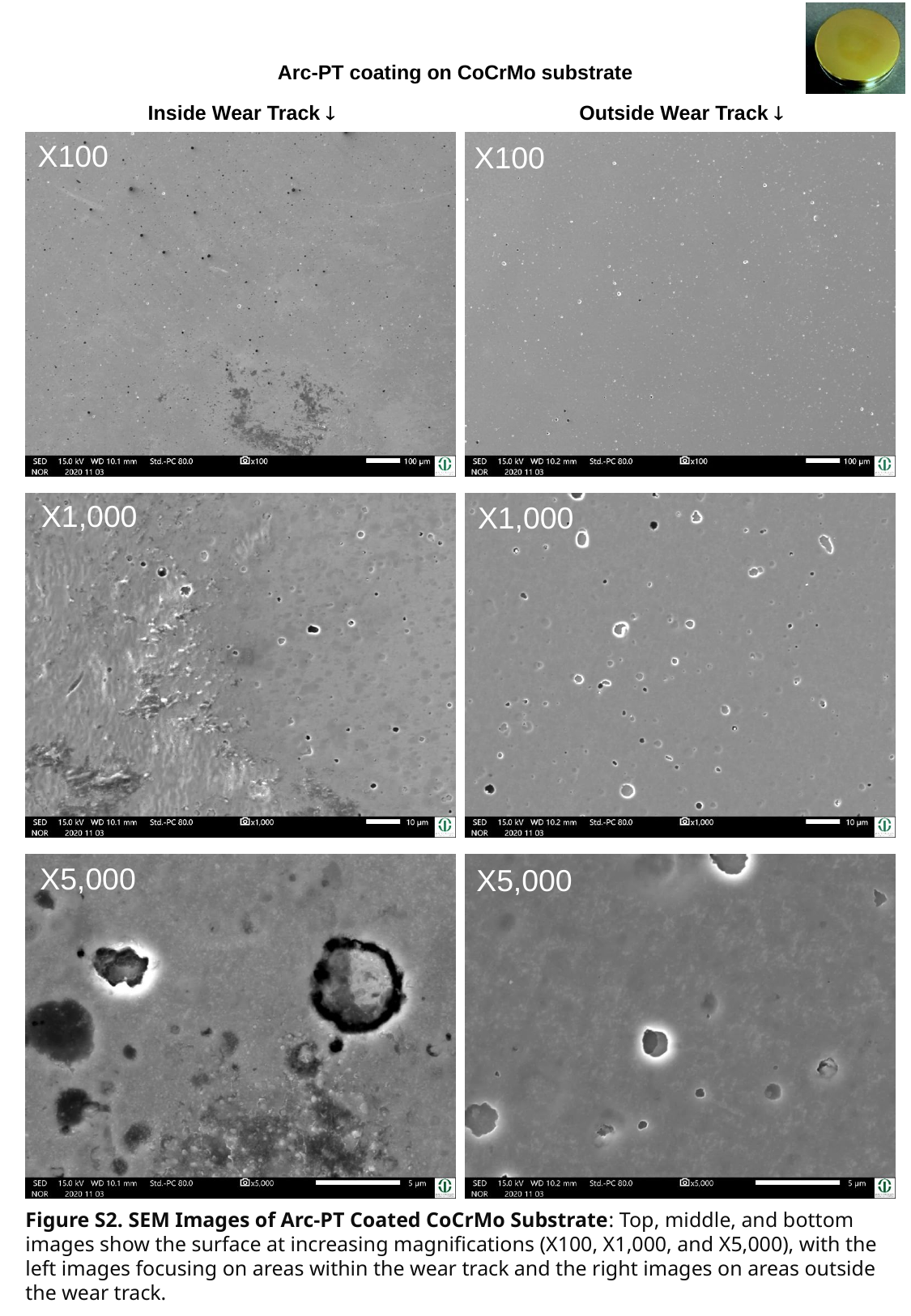

Arc-PT coating on CoCrMo substrate
Inside Wear Track 
Outside Wear Track 
X100
X100
X1,000
X1,000
X5,000
X5,000
Figure S2. SEM Images of Arc-PT Coated CoCrMo Substrate: Top, middle, and bottom images show the surface at increasing magnifications (X100, X1,000, and X5,000), with the left images focusing on areas within the wear track and the right images on areas outside the wear track.

## Slide 3
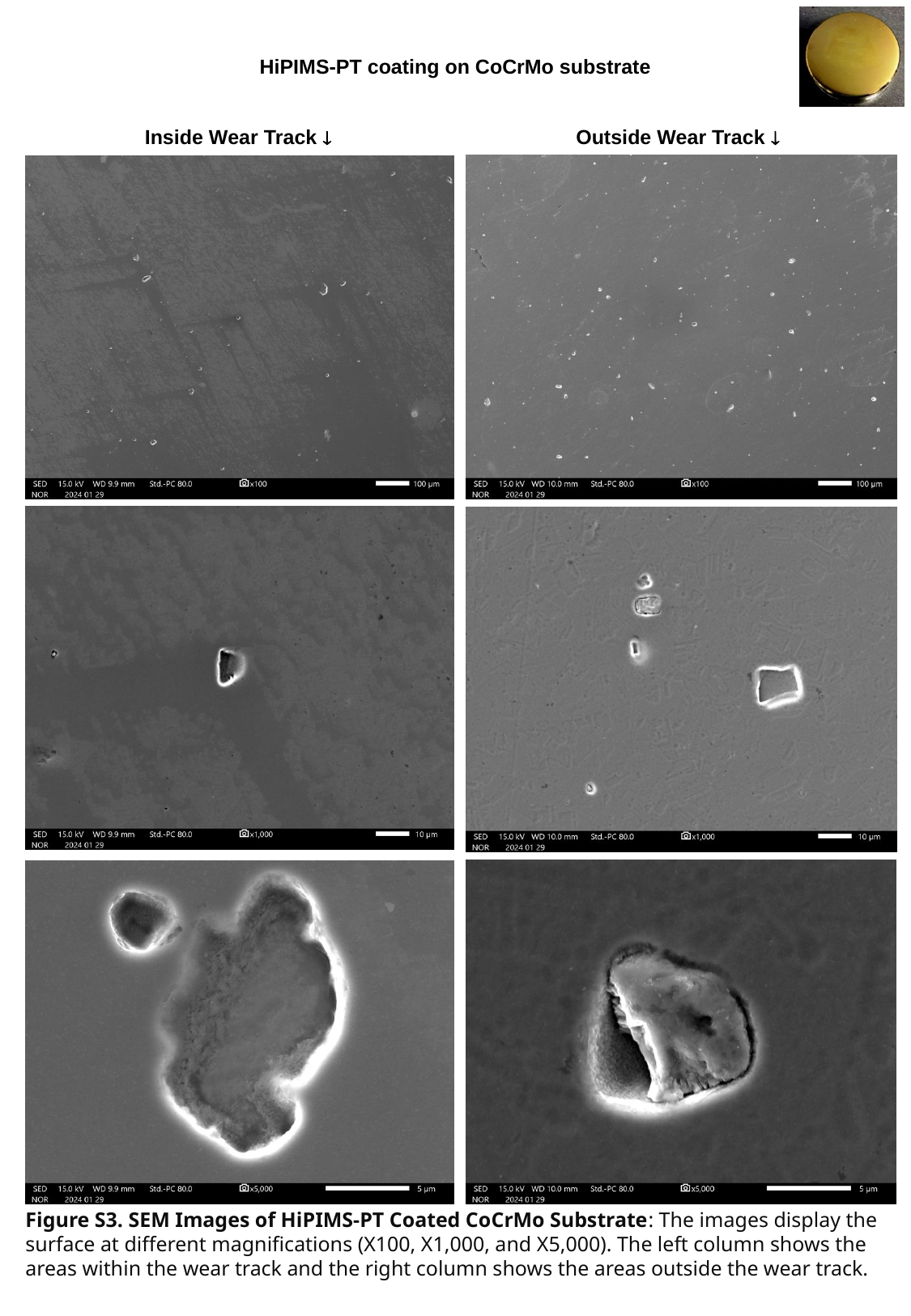

HiPIMS-PT coating on CoCrMo substrate
Inside Wear Track 
Outside Wear Track 
X100
X1,000
X1,000
X5,000
X5,000
Figure S3. SEM Images of HiPIMS-PT Coated CoCrMo Substrate: The images display the surface at different magnifications (X100, X1,000, and X5,000). The left column shows the areas within the wear track and the right column shows the areas outside the wear track.

## Slide 4
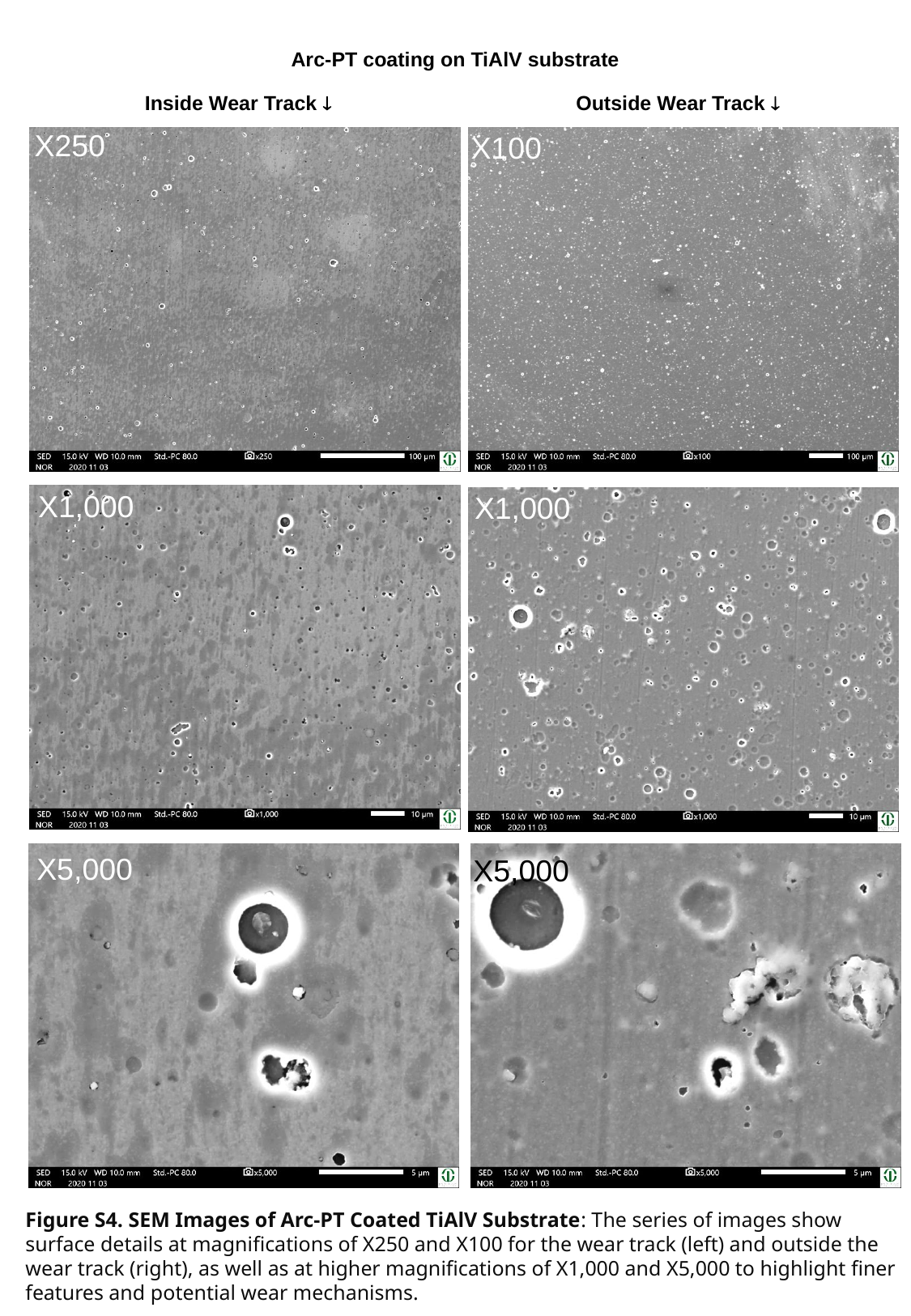

Arc-PT coating on TiAlV substrate
Inside Wear Track 
Outside Wear Track 
X250
X100
X1,000
X1,000
X5,000
X5,000
Figure S4. SEM Images of Arc-PT Coated TiAlV Substrate: The series of images show surface details at magnifications of X250 and X100 for the wear track (left) and outside the wear track (right), as well as at higher magnifications of X1,000 and X5,000 to highlight finer features and potential wear mechanisms.

## Slide 5
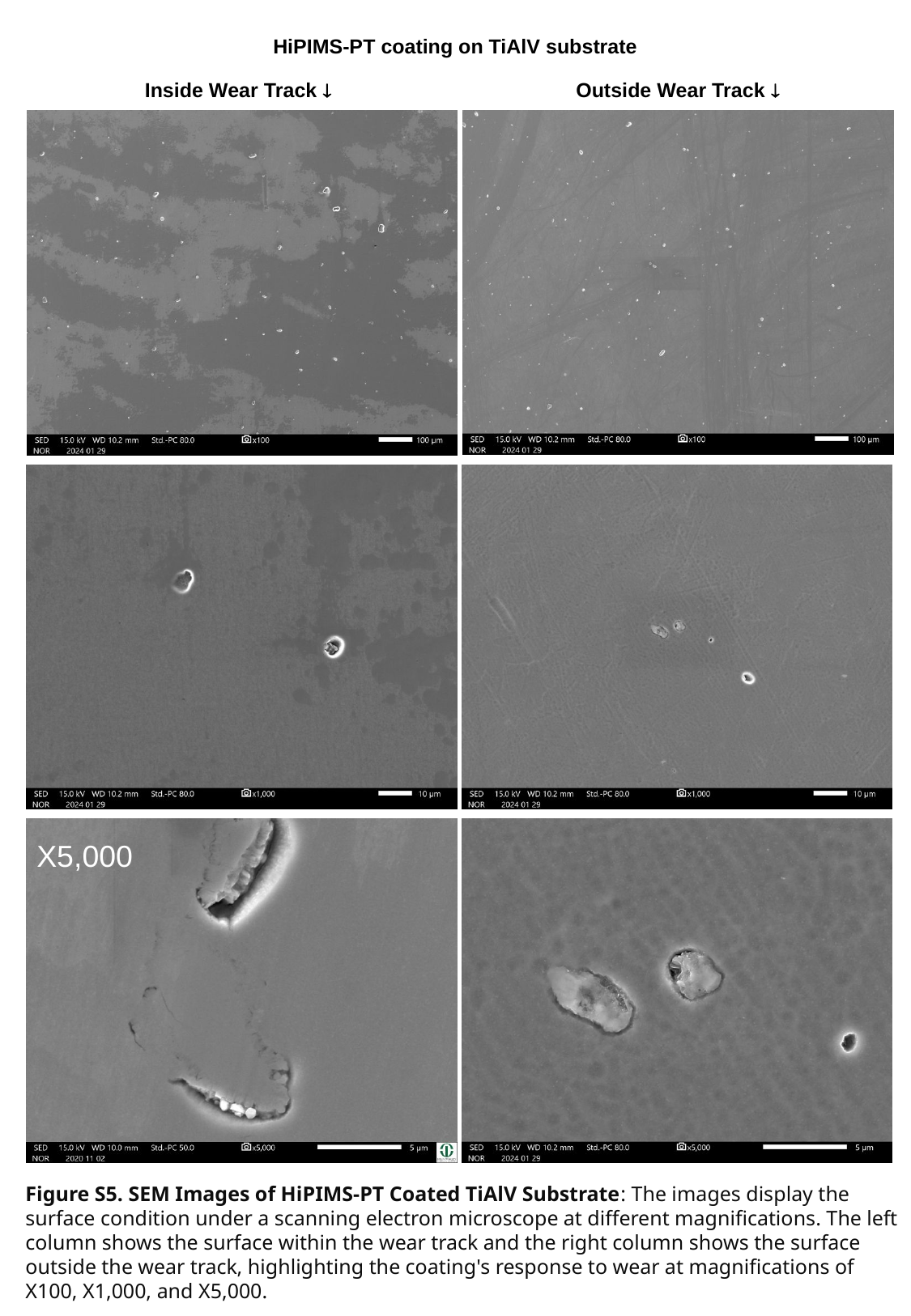

HiPIMS-PT coating on TiAlV substrate
Inside Wear Track 
Outside Wear Track 
X100
X100
X1,000
X5,000
Figure S5. SEM Images of HiPIMS-PT Coated TiAlV Substrate: The images display the surface condition under a scanning electron microscope at different magnifications. The left column shows the surface within the wear track and the right column shows the surface outside the wear track, highlighting the coating's response to wear at magnifications of X100, X1,000, and X5,000.

## Slide 6
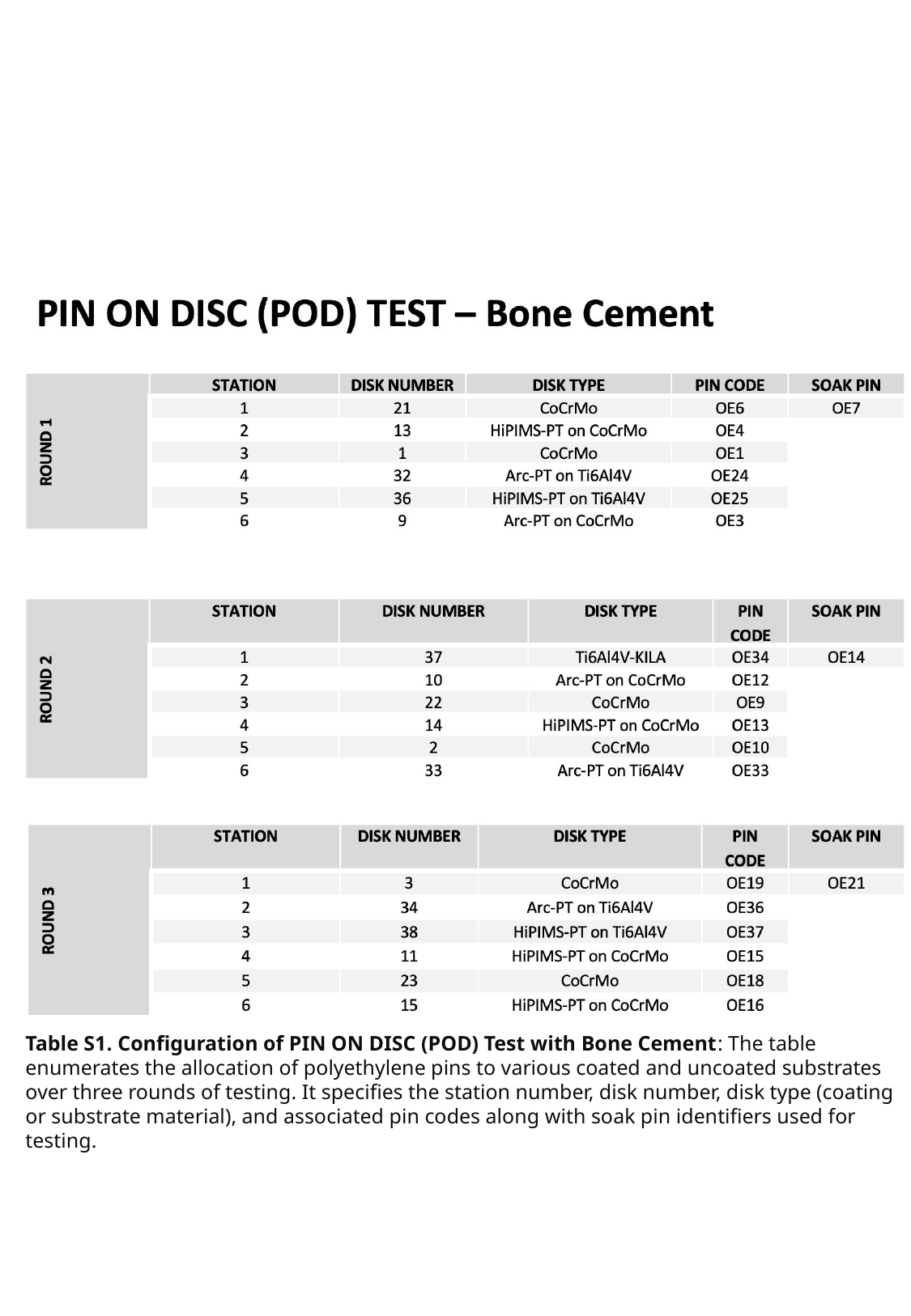

Table S1. Configuration of PIN ON DISC (POD) Test with Bone Cement: The table enumerates the allocation of polyethylene pins to various coated and uncoated substrates over three rounds of testing. It specifies the station number, disk number, disk type (coating or substrate material), and associated pin codes along with soak pin identifiers used for testing.

## Slide 7
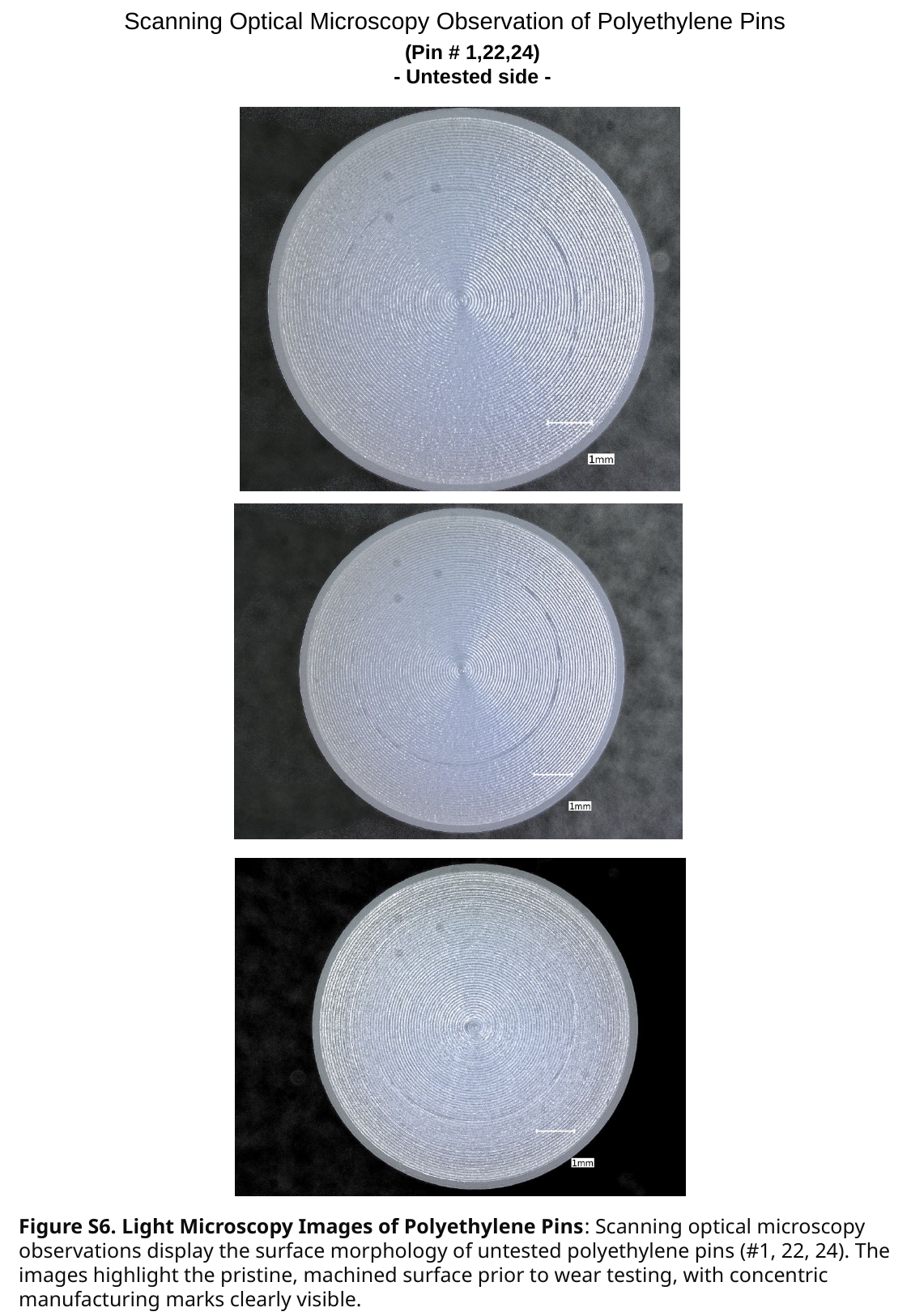

Scanning Optical Microscopy Observation of Polyethylene Pins
(Pin # 1,22,24)
- Untested side -
X100
X100
#1
X1,000
#2
#22
#24
#4
X5,000
X5,000
#3
#34
Figure S6. Light Microscopy Images of Polyethylene Pins: Scanning optical microscopy observations display the surface morphology of untested polyethylene pins (#1, 22, 24). The images highlight the pristine, machined surface prior to wear testing, with concentric manufacturing marks clearly visible.

## Slide 8
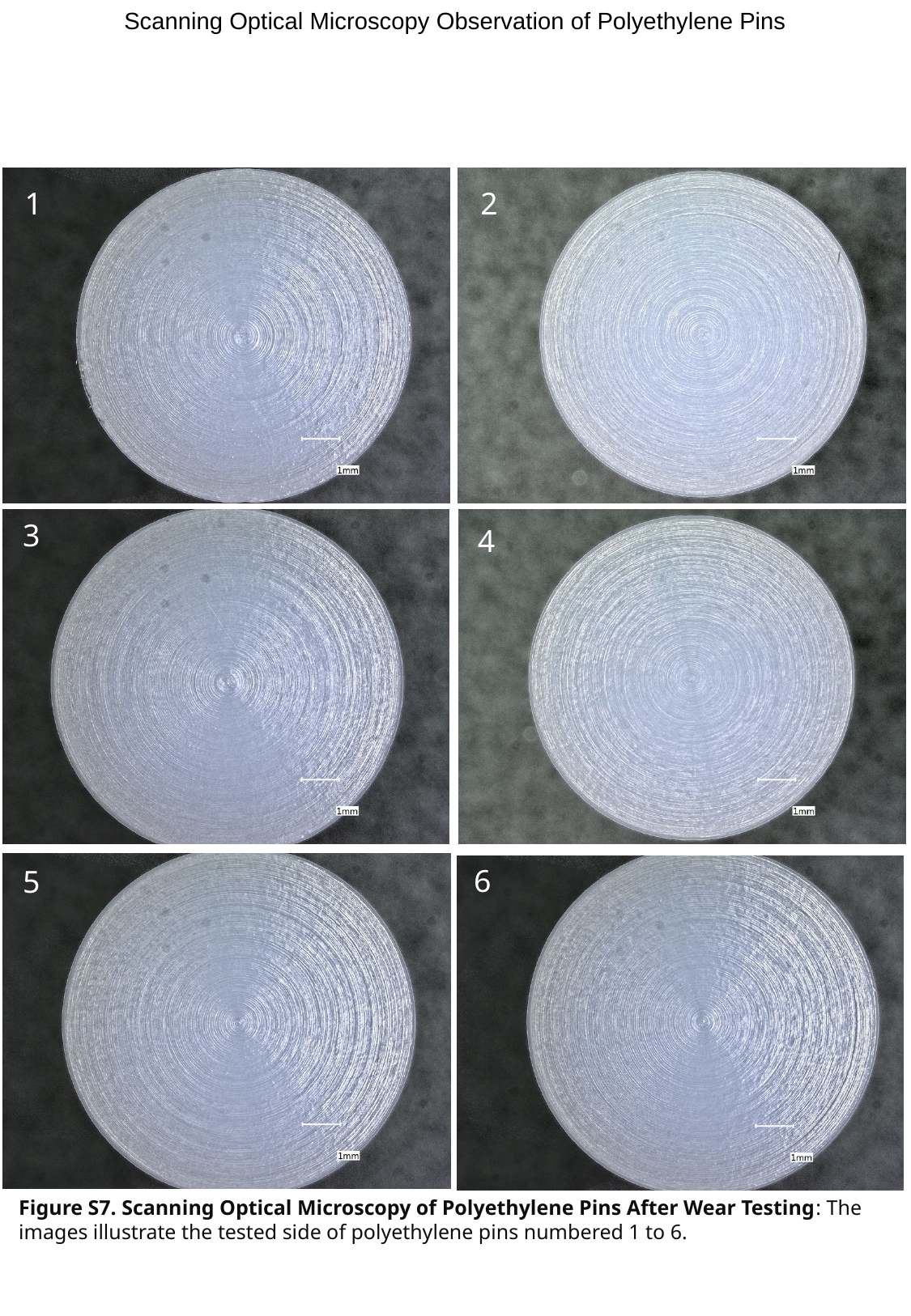

Scanning Optical Microscopy Observation of Polyethylene Pins
X100
X100
#1
1
2
#1
3
X1,000
4
X1,000
#2
#2
6
5
X5,000
X5,000
#3
Figure S7. Scanning Optical Microscopy of Polyethylene Pins After Wear Testing: The images illustrate the tested side of polyethylene pins numbered 1 to 6.

## Slide 9
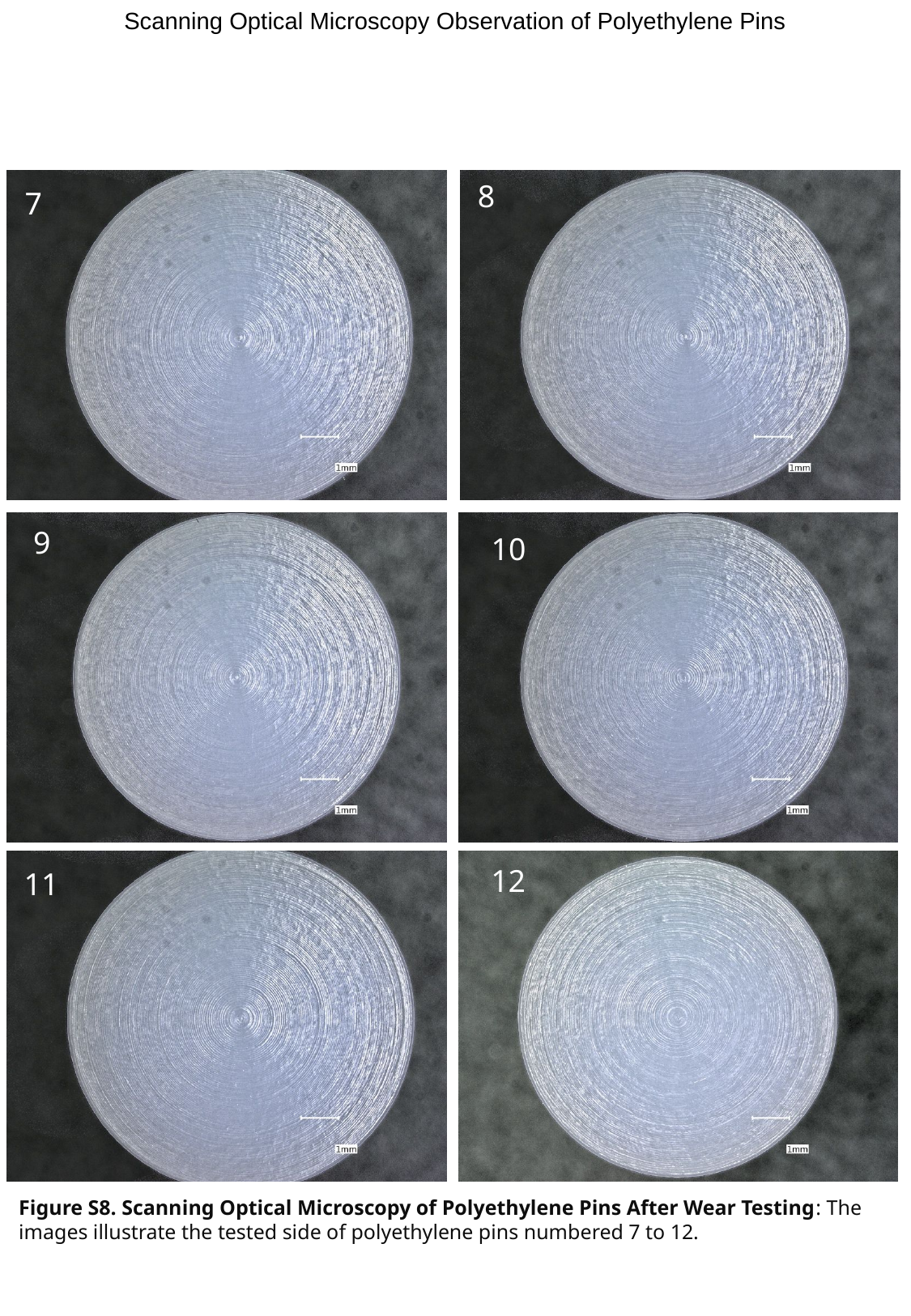

Scanning Optical Microscopy Observation of Polyethylene Pins
X100
X100
8
#1
7
#1
X1,000
9
X1,000
10
#2
#2
12
11
X5,000
X5,000
#3
Figure S8. Scanning Optical Microscopy of Polyethylene Pins After Wear Testing: The images illustrate the tested side of polyethylene pins numbered 7 to 12.

## Slide 10
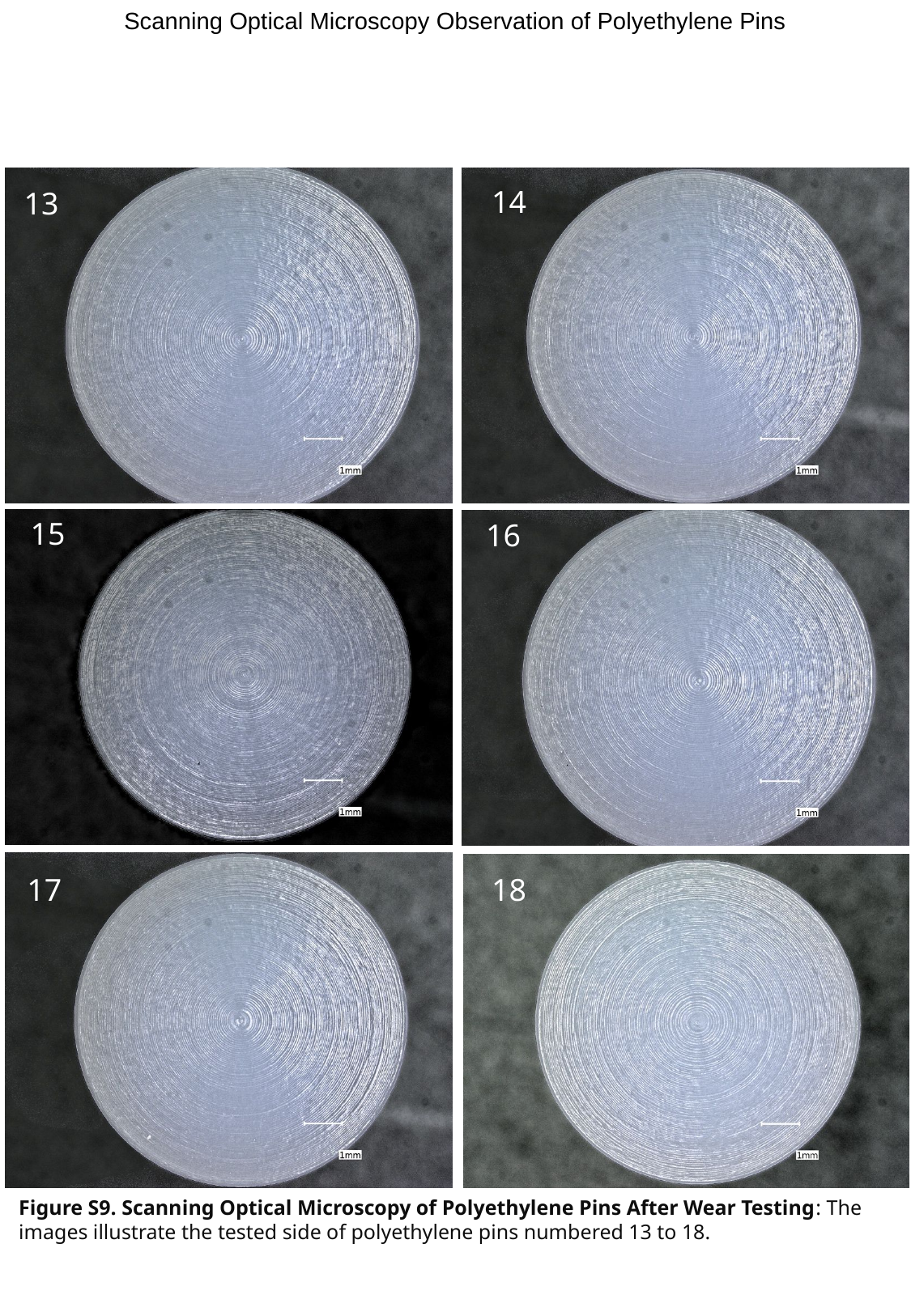

Scanning Optical Microscopy Observation of Polyethylene Pins
X100
X100
#1
14
13
#1
15
16
X1,000
X1,000
#2
#2
17
18
X5,000
X5,000
#3
Figure S9. Scanning Optical Microscopy of Polyethylene Pins After Wear Testing: The images illustrate the tested side of polyethylene pins numbered 13 to 18.

## Slide 11
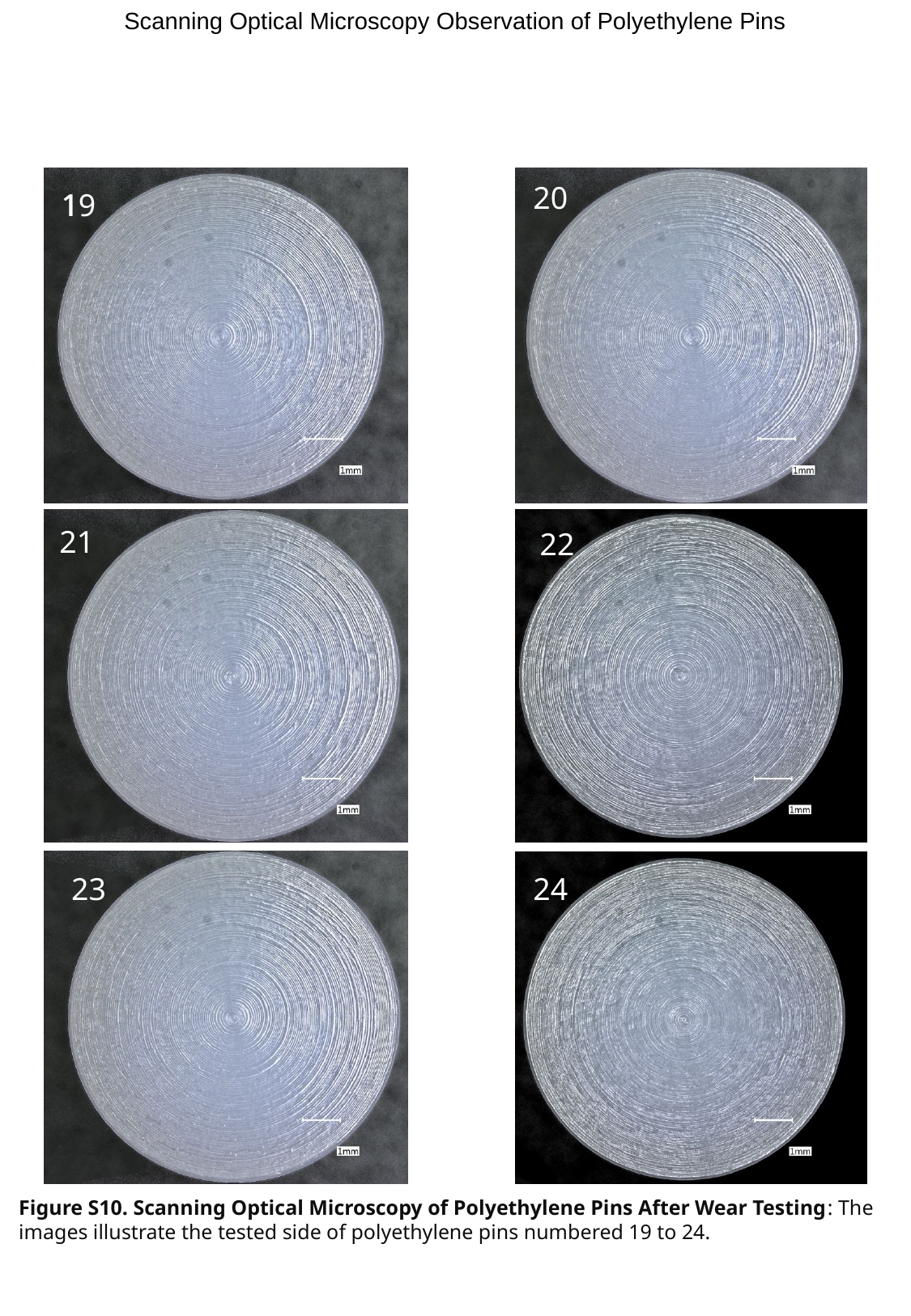

Scanning Optical Microscopy Observation of Polyethylene Pins
X100
X100
20
#1
19
1
#1
21
X1,000
22
X1,000
#2
#2
23
24
X5,000
X5,000
#3
Figure S10. Scanning Optical Microscopy of Polyethylene Pins After Wear Testing: The images illustrate the tested side of polyethylene pins numbered 19 to 24.

## Slide 12
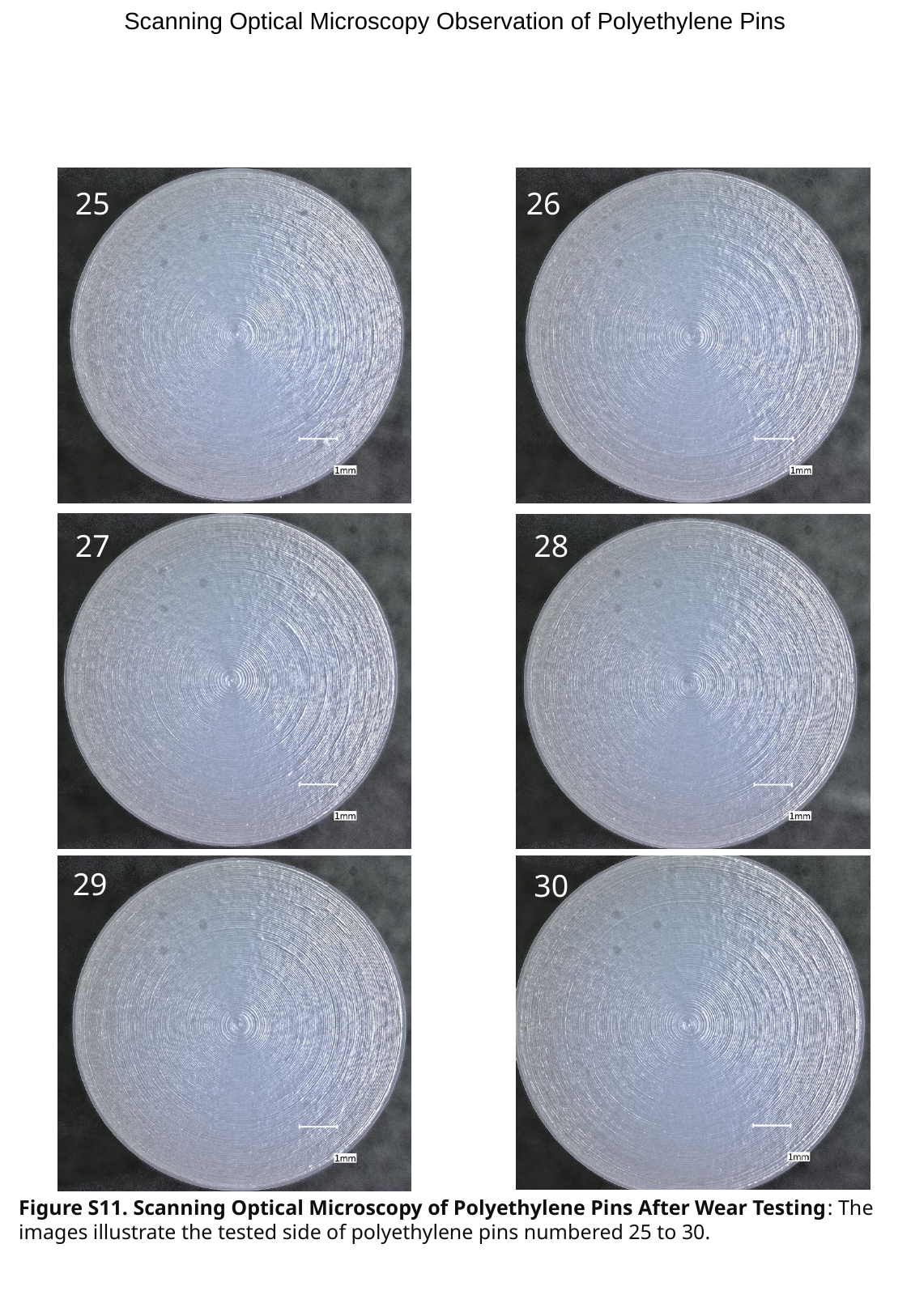

Scanning Optical Microscopy Observation of Polyethylene Pins
25
26
27
28
29
30
Figure S11. Scanning Optical Microscopy of Polyethylene Pins After Wear Testing: The images illustrate the tested side of polyethylene pins numbered 25 to 30.

## Slide 13
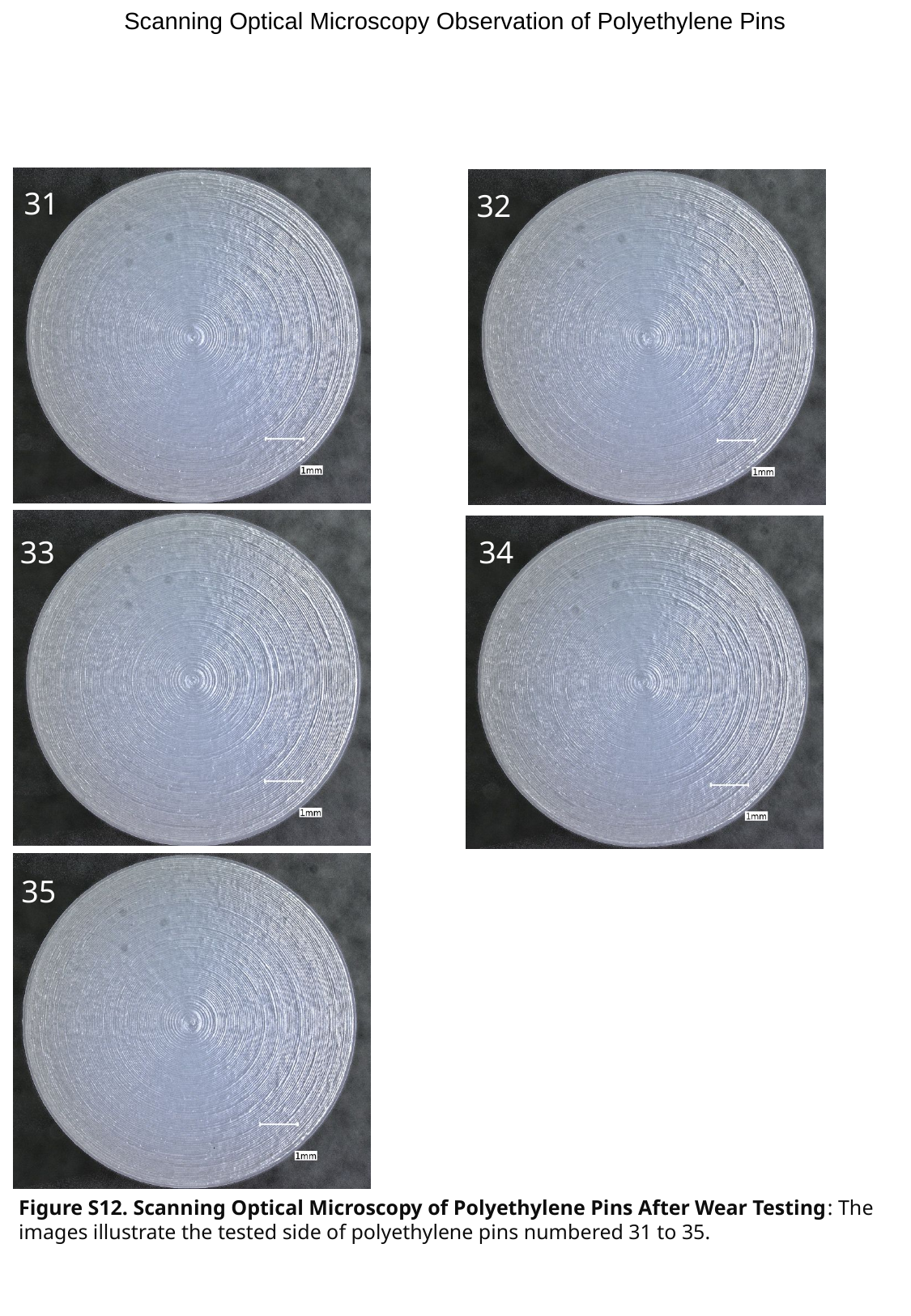

Scanning Optical Microscopy Observation of Polyethylene Pins
25
31
26
32
27
28
33
34
29
30
35
Figure S12. Scanning Optical Microscopy of Polyethylene Pins After Wear Testing: The images illustrate the tested side of polyethylene pins numbered 31 to 35.
